# Programmable Enzymatic Reaction Network in Artificial Cell‐Like Polymersomes

**DOI:** 10.1002/advs.202305760

**Published:** 2024-04-16

**Authors:** Hanjin Seo, Hyomin Lee

**Affiliations:** ^1^ Department of Chemical Engineering Pohang University of Science and Technology (POSTECH) 77 Cheongam‐Ro, Nam‐Gu Pohang Gyeongbuk 37673 South Korea

**Keywords:** artificial cell, cell‐to‐cell communication, coacervates, microfluidics, polymersome

## Abstract

The ability to precisely control in vitro enzymatic reactions in synthetic cells plays a crucial role in the bottom‐up design of artificial cell models that can recapitulate the key cellular features and functions such as metabolism. However, integration of enzymatic reactions has been limited to bulk or microfluidic emulsions without a membrane, lacking the ability to design more sophisticated higher‐order artificial cell communities for reconstituting spatiotemporal biological information at multiple length scales. Herein, droplet microfluidics is utilized to synthesize artificial cell‐like polymersomes with distinct molecular permeability for spatiotemporal control of enzymatic reactions driven by external signals and fuels. The presence of a competing reverse enzymatic reaction that depletes the active substrates is shown to enable demonstration of fuel‐driven formation of sub‐microcompartments within polymersomes as well as realization of out‐of‐equilibrium systems. In addition, the different permeability characteristics of polymersome membranes are exploited to successfully construct a programmable enzymatic reaction network that mimics cellular communication within a heterogeneous cell community through selective molecular transport.

## Graphical abstract

Selective molecular transport as well as signal‐driven formation and dissolution of coacervates have been investigated using artificial cells‐like polymersomes. Programming enzymatic reaction networks in polymersomes with distinctive permeability enabled realization of out‐of‐equilibrium systems through fuel‐driven formation of coacervates followed by autonomous dissolution as well as intercellular communication in heterogeneous cell community.

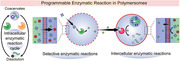

## 1 Introduction

Cells, the essential building blocks of life, can spatiotemporally regulate a series of coupled biochemical reactions to perform a variety of cellular functions as well as complex biochemical tasks.^[^ ^1^, ^2^ ^]^ Among these, cellular metabolism is a set of biochemical reactions that occurs in living organisms to maintain life.^[^ ^2^ ^]^ Such metabolic activity allows the chemical transformation of molecules to provide the energy required for the cell to maintain an out‐of‐equilibrium state and typically involves sequences of enzymatic reactions within the internal milieu as well as controlled transport of cellular signals through the membrane.^[^ ^3^ ^]^ Biological cells orchestrate these signal cascades to communicate with nearby cells and even form internal substructures to dynamically regulate biochemical reactions.^[^ ^1^ ^]^ To mimic these key features using the bottom‐up approach, various types of synthetic cellular membranes and sub‐cellular structures have been integrated into artificial cell models for the realization of biochemical reactions.^[^ ^4^, ^5^, ^6^, ^7^, ^8^, ^9^ ^]^ These artificial cell‐like structures include liposomes,^[^ ^6^ ^]^ surfactant‐stabilized droplets,^[^ ^5^ ^]^ polymersomes,^[^ ^7^ ^]^ colloidosomes,^[^ ^10^ ^]^ proteinosomes,^[^ ^11^ ^]^ coacervate‐core vesicles,^[^ ^12^, ^13^, ^14^ ^]^ and actinosomes,^[^ ^15^ ^]^ of which liposomes and polymersomes have been most widely investigated in remodeling life‐like traits such as cell‐free gene expression,^[^ ^16^ ^]^ cytoskeleton assembly,^[^ ^7^, ^17^ ^]^ endocytosis,^[^ ^18^ ^]^ cell‐cell communication,^[^ ^19^ ^]^ cell growth,^[^ ^20^ ^]^ and division.^[^ ^21^ ^]^ Liposomes prepared from natural lipids that constitute the actual cell membrane exhibit excellent biocompatibility and thus can more effectively mimic the cell membrane without the need to consider compatibility issues with various substances involved in biochemical reactions. However, liposomes often suffer from intrinsic fragility and instability issues which limit their broader applicability.^[^ ^4^ ^]^ These limitations can be mitigated to some extent for polymersomes comprising synthetic amphiphilic block copolymers as they offer enhanced stability and robustness as well as chemical versatility. In particular, polymersomes have been utilized as a promising artificial cell model capable of effectively executing intricate and hierarchical signal cascades akin to cells, owing to their distinct stability and membrane tunability.^[^ ^7^, ^16^, ^19^, ^24^, ^25^, ^26^ ^]^ While such polymersomes hold great promise in mimicking cellular functions, programming biochemical reactions necessitate a precisely controllable bottom‐up approaches to synthesize them. Recently, droplet microfluidics has been employed as an efficient methodology to continuously synthesize polymersomes with high encapsulation efficiency, size monodispersity, and high‐throughput.^[^ ^4^ ^]^ Yet, challenges persist in achieving the requisite spatiotemporal regulation of enzymatic reactions at a sufficient level that can mimic out‐of‐equilibrium systems within polymersomes. While external signal‐driven cascade enzymatic reactions have been demonstrated in these synthetic structures to emulate cellular metabolic pathways,^[^ ^8^, ^27^, ^28^, ^29^ ^]^ further extension to controlling molecular transport within heterogeneous cell communities presents an additional layer of complexity in the development of artificial cell models.

**Scheme 1:**
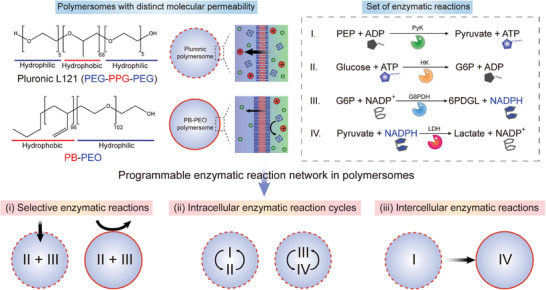
Schematics illustrating the programmability of enzymatic reaction network in microfluidically synthesized polymersomes with distinct molecular permeability. For the model set of enzymatic reactions, four enzymatic reactions based on pyruvate kinase (PyK), hexokinase (HK), glucose‐6‐phosphate dehydrogenase (G6PDH), and lactic dehydrogenase (LDH) were selected in which the first two are involved in phosphorylation of adenosine diphosphate (ADP) and dephosphorylation of adenosine triphosphate (ATP), respectively, while the latter two are related to transfer of a hydride between nicotinamide adenine dinucleotide phosphate (NADP^+^) and its reduced form (NADPH). These four reactions can be coupled in various ways to realize (i) selective enzymatic reactions, (ii) intracellular enzymatic reaction cycles, as well as (iii) intercellular enzymatic reactions in polymersomes.

Herein, we report an artificial cell model based on microfluidically synthesized polymersomes with tunable molecular permeability. Droplet microfluidics provides the means to synthesize these polymersomes with precisely controllable size while allowing highly efficient encapsulation of biological substances with exceptional stability and chemical versatility.^[^ ^9^, ^30^, ^31^, ^32^ ^]^ By taking advantage of these unique features and subsequent integration of sets of model enzymatic reactions, we show the programmability of enzymatic reaction network in polymersomes by demonstrating (i) selective enzymatic reactions, (ii) intracellular enzymatic reaction cycles, and (iii) intercellular enzymatic reactions, as illustrated in **Scheme** **1**. Polymersomes with distinct molecular permeability were shown to grant substrate‐specific response, selective coacervation in only a subset of the population as well as dissolution of coacervate droplets through intercellular communication. Moreover, enzymatic reaction cycles comprising forward and reverse reaction that dynamically regulates the active substrates were incorporated in polymersomes for transient formation and dissolution of complex coacervates driven by external fuels to showcase an out‐of‐equilibrium state in artificial cell models.

## 2 Results and Discussion

### 2.1 Microfluidic Synthesis of Artificial Cell‐Like Polymersomes with Distinct Molecular Permeabilities

Polymersomes consist of an aqueous core surrounded by a bilayer membrane comprising an amphiphilic block copolymer. Unlike conventional methods, droplet microfluidics allows to synthesize polymersomes with an unilamellar structure, high uniformity and encapsulation efficiency through an evaporation‐induced dewetting process that leads to self‐assembly of these amphiphilic block copolymers into a bilayer membrane, as detailed in our previous works.^[^ ^7^, ^31^ ^]^ Briefly, to synthesize these polymersomes, we prepare monodisperse water‐in‐oil‐in‐water (W/O/W) double emulsion droplet templates using glass capillary‐based microfluidic devices (Schemes S1 and S2 and Figures S1 and S2, Supporting Information) and form a polymeric bilayer membrane with various compositions. Two representative model polymersomes with similar sizes (Figure S3, Supporting Information) but exhibiting distinctive permeabilities were produced as illustrated in Scheme 1. One is the Pluronic polymersomes consisting of poly(ethylene glycol)(PEG)_0.2k_‐b‐poly(propylene glycol)(PPG)_3.9k_‐b‐poly(ethylene glycol)(PEG)_0.2k_ (Pluronic L121) with a low hydrophilic‐to‐lipophilic balance (HLB = 7) as well as low hydrophilic‐to‐hydrophobic volume ratio (*f* value) of 0.12. This results in a membrane that is highly permeable to hydrophilic molecules with an estimated molecular weight cut‐off of 500 Da.^[^ ^7^, ^9^ ^]^ The other is the less permeable analogue that comprises of poly(butadiene)‐b‐poly(ethylene oxide) (PB_5.2k_‐PEO_4.5k_). Here, the molecular weight of the hydrophobic blocks is similar, but the PB block is more hydrophobic compared to the PPG block.^[^ ^33^, ^34^ ^]^ Moreover, the higher *f* value (0.86) of PB‐PEO than the Pluronic L121 (0.12) indicates that PB‐PEO polymersomes have a thicker and denser exterior PEO layer than Pluronic polymersomes^[^ ^9^, ^30^, ^31^, ^33^ ^]^; the overall bilayer thickness is also predicted to be thicker for the PB‐PEO polymersomes.^[^ ^9^, ^34^ ^]^

**Figure 1:**
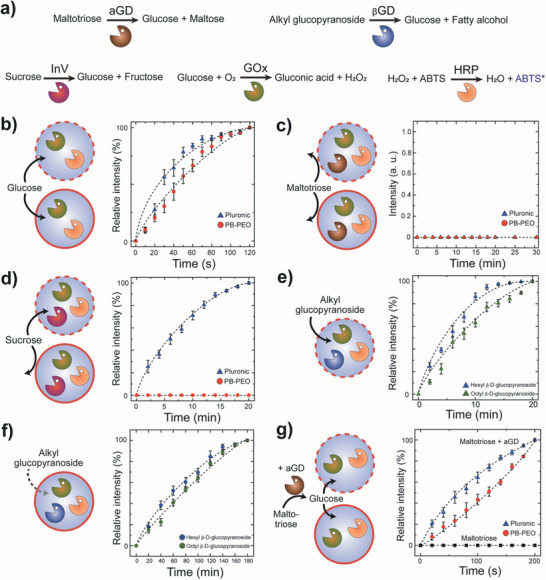
Colorimetric analysis based on cascade enzymatic reactions for determination of the polymersomes’ molecular permeability. a) Sets of enzymatic reactions involving α‐glucosidase (aGD), β‐D‐glucosidase (βGD), InV, GOx, and HRP. b) Schematic illustrating the GOx/HRP double cascade enzymatic reaction driven by glucose signaling and the corresponding plot showing the relative intensity profile with time for Pluronic polymersomes (blue triangle) and PB‐PEO polymersomes (red circle) (*n* = 10). c) Schematic illustrating the aGD/GOx/HRP triple cascade enzymatic reaction driven by maltotriose signaling and the corresponding plot showing the relative intensity profile with time for each polymersome (*n* = 10). d) Schematic illustrating the InV/GOx/HRP triple cascade enzymatic reaction driven by sucrose signaling and the corresponding plot showing the relative intensity profile with time for each polymersome (*n* = 10). e,f) Schematic illustrating the βGD/GOx/HRP triple cascade enzymatic reaction driven by hexyl β‐D‐glucopyranoside (264 Da, blue triangle and circle) and octyl β‐D‐glucopyranoside (292 Da, green triangle and circle) signaling and the corresponding relative intensity profile for each polymersome (*n* = 10). g) Schematic illustrating the GOx/HRP cascade enzymatic reaction by maltotriose signaling before and after post‐injection of aGD and the corresponding relative intensity profile plots for PB‐PEO and Pluronic polymersomes (n = 10). Concentrations of all enzymes are 10 U mL^−1^, and ABTS is 1 mm. All pH conditions are 7.4. Error bars represent standard deviation. All curves are guide‐to‐the‐eye.

To confirm whether PB‐PEO polymersomes (solid circle) are less permeable to hydrophilic molecules than the Pluronic polymersomes (dashed circle), we performed colorimetric analysis based on glucose oxidase (GOx) and horseradish peroxidase (HRP) cascade enzymatic reaction with diffusive glucose (180 Da) signaling and a redox responsive colorimetric dye, 2,2′‐azino‐bis(3‐ethylbenzothiazoline‐6‐sulfonic acid) (ABTS) (**Figure** **1**a). Incorporation of additional enzymes, α‐glucosidase (aGD), β‐D‐glucosidase (βGD), and invertase (InV) into this cascade reaction allows the enzymatic breakdown of di‐ and tri‐saccharide such as sucrose (342 Da) and maltotriose (504 Da) for the investigation of their permeability across each polymersome membrane. In both polymersomes, a clear blue signal resulting from the enzymatic conversion to ABTS radical (ABTS^*^) was observed upon external injection of 50 mm glucose (Figure 1b). Conversely, no noticeable color signal was detected upon injection of 50 mm maltotriose (Figure 1c). Further extending this assay to sucrose (Figure 1d) as well as hexyl‐ and octyl β‐D‐glucopyranoside (264 and 292 Da, Figure 1e,f) revealed that PB‐PEO polymersomes exhibited lower permeability compared to that of Pluronic polymersomes, as evidenced by an order of magnitude increase in the diffusion time scale for both alkyl glucopyranosides; note that hexyl β‐D‐glucopyranoside with lower molecular weight compared to octyl β‐D‐glucopyranoside exhibited slightly faster permeation rate for both polymersomes as the differences in enzymatic reaction rates were not significant (Figure S4, Supporting Information). We also observed that while both polymersomes do not allow permeation of maltotriose, subsequent post‐injection of aGD in the media which converts maltotriose into a diffusive glucose signal leads to clear detection of blue signals in both cases (Figure 1g). Moreover, an additional injection of a reducing agent, dithiotreitol (DTT, 154 Da) was shown to effectively permeate through the membrane and convert the ABTS^*^ (blue signal) into colorless ABTS (Figure S5, Supporting Information). These results indicate that PB‐PEO polymersomes enable permeation of these neutral molecules close to 300 Da, whereas Pluronic polymersomes allow passage of up to 500 Da at pH 7.4.

However, as many of the enzymatic substrates are charged in physiological pH conditions (pH 7.4), it is imperative to further extend to molecules with charges. Additional colorimetric analysis on the permeability of negatively charged reducing agents and enzymatic substrates relevant to this work revealed that Pluronic polymersomes are permeable toward glutathione (GSH, 307 Da, charge of the component at pH 7.4 = ‐e,^[^ ^35^ ^]^ Figure S6a, Supporting Information), glucose‐6‐phosphate (G6P, 260 Da, −2e,^[^ ^36^ ^]^ Figure S7a, Supporting Information), phosphoenol pyruvate (PEP, 180 Da, −3e,^[^ ^37^ ^]^ Figure S8a, Supporting Information), and pyruvate (88 Da, ‐e,^[^ ^38^ ^]^ Figure S9a, Supporting Information) at pH of 7.4, regardless of their charge, which is consistent with our previous report.^[^ ^7^ ^]^ On the other hand, we found that PB‐PEO polymersomes only allowed pyruvate to permeate through among all negatively charged molecules tested (Figures S6–S9b and Table S1, Supporting Information). This behavior is attributed to the more hydrophobic nature of the PB block compared to the PPG block which requires higher de‐solvation energy for these charged molecules to permeate through the bilayer membrane.^[^ ^33^, ^34^ ^]^ Overall, these results confirm our initial hypothesis that PB‐PEO polymersomes are indeed less permeable to hydrophilic molecules than Pluronic polymersomes.

### 2.2 Substrate‐Specific Response via Selective Enzymatic Reactions in Polymersomes

**Figure 2:**
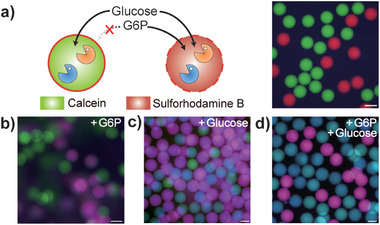
Selective enzymatic reactions in heterogeneous cell populations. a) Schematic illustration and the fluorescence micrograph of the HK and G6PDH double cascade reaction and the polymersomes with distinctive permeability toward G6P. For visualization purposes, PB‐PEO and Pluronic polymersomes were each labelled with calcein (green) and sulforhodamine B (red), respectively. b–d) Fluorescence micrographs showing the substrate‐specific colorimetric response in a heterogeneous polymersome population with distinct molecular permeability. Scale bars represent 100 µm. Concentrations of calcein and sulforhodamine B are fixed at 5 µg mL^−1^. Fluorescence micrographs were acquired 4 hr after injection of each substrate into the media.

The ability to synthesize sets of polymersomes with distinct permeability toward enzymatic substrates offers new opportunities for selectively inducing enzymatic reactions within a heterogeneous cell community. To achieve this, we integrate a double cascade enzymatic reaction based on hexokinase (HK, reaction II in Scheme 1) and glucose‐6‐phosphate dehydrogenase (G6PDH, reaction III in Scheme 1) into both PB‐PEO and Pluronic polymersomes. We differentiate these two sets of polymersomes by incorporating non‐permeable fluorescent dyes: calcein (green) for PB‐PEO and sulforhodamine B (red) for Pluronic polymersomes, as shown in the schematics and fluorescence micrographs of **Figure** **2**a. The first reaction based on HK catalyzes the phosphorylation of glucose by adenosine triphosphate (ATP) to glucose‐6‐P (G6P) and the second reaction involving G6PDH converts G6P into 6‐phosphoglucono‐δ‐lactone (6PGDL) while oxidizing the colorless nicotinamide adenine dinucleotide phosphate (NADP^+^) into a blue, fluorescent reduced form, NADPH. As a result, the execution of the second enzymatic reaction based on G6PDH can be easily visualized within this heterogeneous population. In the presence of excess ATP and NADP^+^ within both sets of polymersomes, we observed selective permeation of injected G6P into the Pluronic polymersomes, followed by emission of blue fluorescence signal from NADPH, altering the initially red‐colored polymersomes into magenta (Figure 2b). Additional glucose injection into the media led to activation of both sets of polymersomes, as evidenced by the appearance of cyan‐colored PB‐PEO polymersomes and saturation level increase for the magenta‐colored, Pluronic polymersomes compared to the ones exposed only to glucose without additional G6P (Figure 2c,d). These results clearly demonstrate that the distinct molecular permeability of polymersomes can be exploited to selectively induce enzymatic reactions and achieve substrate‐specific responses in heterogeneous cell communities.

### 2.3 Signal‐Driven Formation and Dissolution of Coacervates in Bulk via Enzymatic Reactions

**Figure 3:**
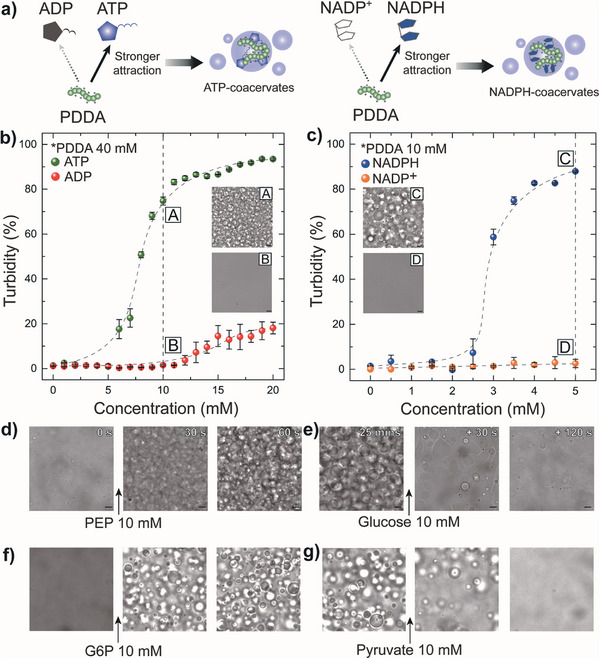
Signal‐driven formation and dissolution of coacervates in bulk. a) Schematics illustrating the preferential interaction of ATP and NADPH with PDDA that leads to formation of ATP‐ and NADPH‐coacervates, respectively. b) Turbidity plot with respect to ADP and ATP concentrations at a fixed PDDA concentration of 40 mm (*n* = 5). c) Turbidity plot with respect to NADPH and NADP^+^ concentrations at a fixed PDDA concentration of 10 mm (*n* = 5). All curves are guide‐to‐the‐eye, and the error bars indicate standard deviations. The inset optical micrographs show the mixture at defined condition and scale bars represent 10 µm. d) Series of optical micrographs depicting the formation of ATP‐coacervates in bulk upon introducing 10 mm PEP. Concentration of ADP and PDDA in the media is 10 mm and 40 mm, respectively. e) Series of optical micrographs depicting the dissolution of ATP‐coacervates in bulk upon introducing 10 mm glucose. f) Series of optical micrographs depicting the formation of NADPH‐coacervates in bulk upon introducing 10 mm G6P. Concentration of NADP^+^ and PDDA in the media is 5 mm, and 10 mm, respectively. g) Series of optical micrographs depicting the dissolution of NADPH‐coacervates in bulk upon introducing 10 mm pyruvate. All scale bars represent 10 µm and the concentration of enzymes is fixed at 10 U mL^−1^ and the pH is set at 7.4.

We further extended this concept to spatiotemporally regulate coacervation within polymersomes. Coacervates are formed by associative liquid‐liquid phase separation between complementarily interacting macromolecules that include small molecules, metal ions, polymers, and proteins. In particular, small multivalent anionic molecules can complex with complementary cationic counterparts through electrostatic attraction, leading to spontaneous formation of coacervate droplets via phase separation from the surrounding media.^[^ ^4^ ^]^ These complex coacervates have been shown to exhibit high mass transfer rate as well as selective partitioning tendencies, providing the means to spatiotemporally coordinate biochemical reactions in artificial cell‐like structures.^[^ ^39^ ^]^ To exploit this potential, we first chose two metabolites, ATP and NADPH, which are fundamental enzymatic substrates and serve as cellular energy currencies as the anionic molecules.^[^ ^40^ ^]^ Poly(diallyl dimethyl ammonium chloride) (PDDA) was selected as the common complementary polycation that interacts with these anionic molecules. Here, the key design principle is to dynamically modulate the multivalency of these anionic biomolecules through enzymatic reactions, thereby controlling the electrostatic interactions with its cationic counterpart, PDDA, to either induce formation or dissolution of coacervates. The formation and dissolution of these two sets of complex coacervates, ATP‐ and NADPH‐coacervates, were demonstrated by utilizing enzymatic reactions based on pyruvate kinase (PyK, reaction I in Scheme 1) and G6PDH (reaction III in Scheme 1) that each produces ATP and NADPH, respectively, as well as their complementary reverse enzymatic reactions based on HK (reaction II in Scheme 1) and lactic dehydrogenase (LDH, reaction IV in Scheme 1) that consumes these substrates. As ATP possesses an additional phosphate group compared to ADP, and NADPH exhibits larger negative charge than its oxidized form, NADP^+^, these sets of enzymatic reactions can be exploited to control the strength of electrostatic attraction with its counterpart, PDDA, to induce complex coacervation,^[^ ^40^ ^]^ as illustrated in **Figure** **3**a. To determine the condition at which this enzymatic conversion of the substrate leads to formation of ATP‐ and NADPH‐coacervates, we performed turbidity assay in bulk for both nucleotides, ADP/ATP, as well as NADP^+^/NADPH. We found that ATP exhibited a noticeable formation of distinct coacervate droplets with PDDA at concentrations above 10 mm, while ADP with one less phosphate group than ATP did not (Figure 3b). Likewise, NADPH also showed a similar behavior where NADPH formed coacervates with PDDA above 3 mm in concentration whereas NADP^+^ did not (Figure 3c). Leveraging on these selective coacervate formation behaviors, we first implemented external signal‐driven, coacervate formation in bulk. The enzymatic formation and dissolution of ATP‐coacervates have been confirmed by sequential injection of PEP and glucose, which each serves as substrates for PyK and HK, respectively (Figure 3d,e). Similarly, the NADPH‐coacervates were also shown to form and dissolve through the injection of G6P and pyruvate in the presence of G6PDH and LDH (Figure 3f,g). Prior to demonstrating the applicability of this concept to two sets of polymersomes with distinctive permeability described earlier, we performed additional turbidity assays to rule out the effect of other substrates with phosphate groups (PEP and G6P) on coacervation (Figure S10a,b, Supporting Information). We confirmed a slight turbidity increase above 20 mM for PEP, while G6P had no effect on coacervate formation. Thus, to mitigate the potential interference of PEP on ATP‐coacervate formation while simultaneously acquiring immediate response upon enzymatic conversion into ATP, we set the post‐injection concentration of PEP at 15 mm (Figure S10c, Supporting Information). Also, the effect of enzymatic reaction byproducts was additionally investigated and were shown to have negligible impact on the turbidity changes associated with both ATP‐ and NADPH‐coacervates (Figure S11, Supporting Information).

### 2.4 Signal‐Driven Formation and Dissolution of Coacervates in Polymersomes via Selective Enzymatic Reactions

**Figure 4:**
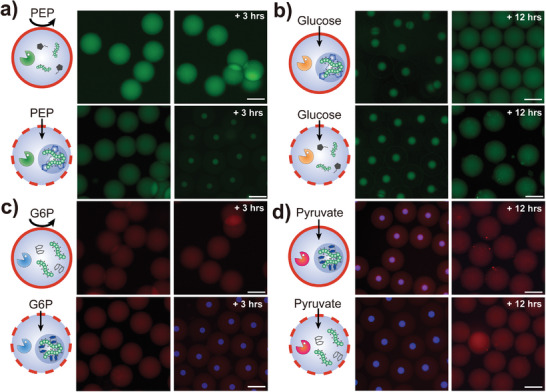
a,b) Schematics and fluorescence micrographs showing the selective signal‐driven formation and dissolution of ATP‐coacervates in polymersomes. a) Concentration of ADP is set at 10 mm and PEP is post‐injected to make up 15 mm in total concentration. b) Concentration of ATP is set at 10 mM and glucose is post‐injected to make up 50 mm in total concentration. c,d) Schematics and fluorescence micrographs showing the selective signal‐driven formation and dissolution of NADPH‐coacervates in polymersomes. c) Concentration of NADP^+^ is set at 5 mm and G6P is post‐injected to make up 20 mm in total concentration. d) Concentration of NADPH is set at 5 mm and pyruvate is post‐injected to make up 50 mm in total concentration. All scale bars in the fluorescence micrographs represent 100 µm.

Building upon our prior demonstration of substrate‐specific enzymatic reaction in polymersomes with distinct permeability, we employed these enzymatic reactions involving ATP and NADPH to achieve spatiotemporal control over the formation and dissolution of distinct coacervates within PB‐PEO and Pluronic polymersomes (**Figure** **4**). For clear visualization of the external signal‐driven formation and dissolution of ATP‐coacervates in both polymersomes, we additionally incorporated 0.1 mg mL^−1^ of fluorescein isothiocynate‐dextran (FITC‐DEX, 70 kDa, green) that exhibit high partitioning tendency (K_p_ ≈53) for ATP‐coacervates. Meanwhile, for NADPH‐coacervates, 0.1 mg mL^−1^ of rhodamine isothiocynate‐dextran (RITC‐DEX, 70 kDa, red) with low K_p_ values (≈1.5) were added in both polymersomes as NADPH is intrinsically fluorescent (blue) (Figure S12, Supporting Information). Upon injection of PEP in the media, we observed formation of ATP‐coacervate droplets only in Pluronic polymersomes which is attributed to the selective diffusion of PEP followed by PyK enzymatic reaction (Figure 4a) as PB‐PEO polymersomes are impermeable to PEP (Figure S8, Supporting Information). Meanwhile, as both polymersomes are permeable toward glucose, pre‐formed ATP‐coacervates in both polymersomes (see Experimental Sections 1.3 and 1.4, Supporting Information for details) dissolved by external glucose‐driven HK enzymatic reaction, as evidenced by the transition from a discrete small droplet to less intense but larger homogeneous fluorescent signal throughout the polymersomes (Figure 4b). Similar tendency was observed for the NADPH‐coacervates in which only Pluronic polymersomes responded to G6P exposure, yielding NADPH‐coacervates (Figure 4c; Figure S7, Supporting Information) while both polymersomes allowed pyruvate to permeate into the polymersomes containing pre‐formed NADPH‐coacervates, leading to dissolution by pyruvate‐driven LDH enzymatic reaction (Figure 4d). Overall, these results indicate that we can use external chemical signals to selectively induce either formation or dissolution of coacervates by taking advantage of the variance in the permeability exhibited by these polymersomes.

### 2.5 Fuel‐Driven Regulation of Dissipative Coacervates in Bulk via Enzymatic Reaction Cycles

Up to now, we have shown either the formation or the dissolution of coacervate droplets in polymersomes driven by external signals. However, integrating an enzymatic reaction cycle that comprise of two coupled enzymatic reactions that not only produces but also consumes the identical enzymatic substrate provides the means to dynamically control the formation and dissolution of coacervates in polymersomes. In fact, these enzymatic reaction cycles based on ATP and NADPH resemble the metabolic activities in biological cells to maintain out‐of‐equilibrium state as cells continuously convert energy through both enthalpic interactions and entropic effects.^[^ ^40^, ^41^ ^]^ Synergistically combining such dissipative processes with complex coacervates allows to actively regulate biochemical reactions in a more spatiotemporal manner than equilibrated states due to its adaptability to change in the surrounding environment.^[^ ^42^, ^43^, ^44^, ^45^ ^]^

**Figure 5:**
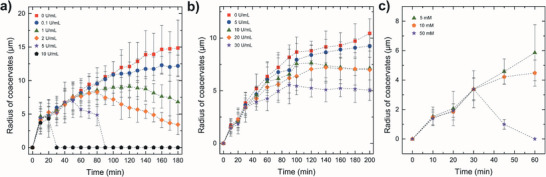
Fuel‐driven dissipative coacervates in bulk by varying the concentrations of either the enzyme or the substrate. a) Plot of time‐dependent ATP‐coacervate droplet size with variation in HK concentration. Concentrations of ADP and PDDA are 10 mm and 40 mm, respectively. Concentrations of PyK, glucose, and PEP (fuel) are fixed at 20 U mL^−1^, 10 mm, and 5 mm, respectively (*n* = 10). b) Plot of time‐dependent NADPH‐coacervate droplet size with variation in LDH concentration. Concentration of G6PDH, pyruvate, and G6P (fuel) are fixed at 20 U mL^−1^, 5 mm, and 3 mm, respectively (*n* = 10). c) Plot of time‐dependent NADPH‐coacervate droplet size with variation in pyruvate concentration. Concentration of G6PDH, LDH, and G6P (fuel) are set as 20 U mL^−1^, 10 U mL^−1^, and 3 mm (*n* = 10). Concentration of NADP^+^ and PDDA are 5 mm and 10 mm, respectively, in both b) and c). All error bars represent standard deviations. All pH conditions are 7.4.

To demonstrate this, we devised out‐of‐equilibrium systems based on dissipative ATP‐ and NADPH‐coacervates where aliquot amount of injected PEP and G6P that each serves as the fuel source initially induces coacervation but soon autonomously dissolves by the dominant reverse reactions, as schematically highlighted in Figure S13 (Supporting Information). We first investigated the ATP‐coacervate dynamics in bulk to explore the interplay between ATP production and consumption through an enzymatic reaction network comprising of PyK and HK. We varied the HK concentration while remaining others constant to determine the role of enzyme concentration ratio in dissipative ATP‐coacervate dynamics (Figure S14, Supporting Information). As the rate of enzyme‐mediated formation and dissolution of coacervates is governed by the relevant enzyme concentrations,^[^ ^46^ ^]^ we initially set the PyK concentration higher than that of HK to generate sufficient ATP for coacervate formation while systematically increasing the HK concentration to study its impact on the coacervate dynamics. Intriguingly, increasing the HK concentration up to 2 U mL^−1^ did not trigger any noticeable dissolution of the ATP‐coacervate within 90 min (**Figure** **5**a). However, at 5 U mL^−1^ HK, a clear dissipative behavior was observed, exhibiting shorter lifetime with further increase in concentration. These findings suggest that the delicate balance between these two complementary enzymatic reactions determine the net ATP accumulation rate thereby enabling dynamic control over the fate of coacervate droplets. Likewise, the same concept was also extended to NADPH‐coacervates where the concentration of LDH was varied while all others were set constant (Figure 5b; Figure S15, Supporting Information). However, unlike the ATP‐coacervates, NADPH‐coacervates did not show a clear indication of dissipative behavior after 200 mins even when the LDH concentration was increased up to 30 U mL^−1^. As a result, we instead varied the reverse reaction enzymatic substrate, pyruvate, concentration from 5 to 10 to 50 mm at a fixed LDH concentration of 10 U mL^−1^ to facilitate the dissolution process. Indeed, we observed a clear dissipative behavior within 60 min upon injection of 50 mM pyruvate as shown in the plot of Figure 5c and the series of optical micrographs shown in Figure S16 (Supporting Information). Here, the weak dependence of NADPH‐coacervate dissolution on LDH concentration compared to analogous HK concentration in ATP‐coacervate is presumably due to the relatively low affinity of LDH for NADPH. The coacervation formation and dissolution behavior is known to be governed by the enzyme concentration as well as the substrate affinity since enzymes with lower affinity for their substrates (high K_M_ value) require higher concentration of substrates for equivalent enzymatic activity.^[^ ^47^ ^]^ In fact, G6PDH that produces NADPH and induces coacervate formation exhibits high affinity for G6P and NADP^+^ (K_M_ values for G6P and NADP^+^ are ≈2.0 × 10^−5 ^M and ≈2.0 × 10^−6 ^M, respectively),^[^ ^48^ ^]^ while LDH involved in the consumption has relatively low affinity for NADPH (≈4.55 × 10^−3 ^M) and high affinity toward pyruvate (K_M_ value ≈ 2.0 × 10^−5 ^M).^[^ ^48^, ^49^, ^50^, ^51^ ^]^ This is consistent with our observation that the NADPH‐coacervate dissolution behavior is more sensitive to the changes in pyruvate concentration compared to changes in that of LDH.^[^ ^52^, ^53^ ^]^ Moreover, considering that our enzymatic network design incorporates two competing enzymes, LDH and G6PDH, the substantial difference in affinity for NADPH and NADP^+^ may further amplify this tendency; the three orders of magnitude difference in affinity of G6PDH for NADP^+^ compared to LDH for NADPH suggests a significantly faster NADPH production rate than consumption. As a result, even in the presence of excess LDH, consumption rate increase may be outpaced by the production through G6PDH, leading to suppression of coacervate dissolution. This notion is further supported by the observed dependence of ATP‐coacervate dynamics on HK concentration which aligns well with our hypothesis regarding substrates affinities to each enzyme. As the reported K_M_ value of PyK to ADP is ≈3.0 × 10^−5 ^M while HK exhibits K_M_ value of ≈ 2.0 × 10^−5 ^M for ATP,^[^ ^54^, ^55^, ^56^, ^57^, ^58^, ^59^, ^60^, ^61^ ^]^ such marginal difference in affinity for ADP and ATP between PyK and HK hints strong dependence of ATP‐coacervate dynamics on the enzyme concentration. Overall, our results demonstrate that spatiotemporal control of coacervate dynamics can be achieved by programming enzymatic reaction cycles with systematic variation in enzyme and substrate concentration.

### 2.6 Fuel‐Driven Regulation of Dissipative Coacervates in Polymersomes via Enzymatic Reaction Cycles

**Figure 6:**
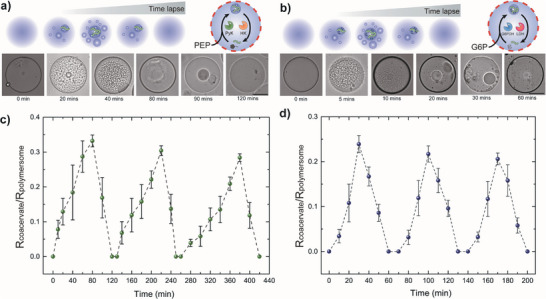
Fuel‐driven dissipative coacervates in polymersomes. a,b) Schematics and time‐lapse optical micrographs showing a) PEP‐driven dissipative ATP‐coacervates in Pluronic polymersomes, and b) G6P‐driven dissipative NADPH‐coacervates in Pluronic polymersomes. Scale bars represent 100 µm. c) Plot of temporal change in the ratio of ATP‐coacervate to polymersome radius upon repeated injection of 5 mm PEP (*n* = 5). d) Plot of temporal change in the ratio of NADPH‐coacervate to polymersome radius upon repeated injection of 3 mm G6P (*n* = 5).

Next, we separately encapsulated the constituents responsible for ATP‐coacervates in one set of Pluronic polymersomes and NADPH‐coacervates in the other followed by subsequent injection of the corresponding fuels into their periphery. In both cases, we successfully demonstrated the fuel‐driven dissipative coacervates in polymersomes where the formed coacervate autonomously dissolved without the need for manual post‐injection of the substrates responsible for the reverse enzymatic reaction. (**Figure** **6**a,b). In addition, we also monitored the ratio of coacervate droplet to polymersome radius upon repeated injection of either PEP or G6P in identical concentration for ATP‐ and NADPH‐coacervates, respectively (Figure 6c,d). We observed that the fuel‐driven dissipative coacervation in polymersome is reversible with the size of the resulting transient coacervate droplet slightly decreasing with repeated cycles, possibly due to the presence of a membrane that affects the mass transport of fuels. Moreover, the polymersome membrane can also cause delay in the coacervate growth rate compared to bulk. While fuel injection in bulk systems results in immediate coacervate formation and subsequent growth, coacervation within polymersomes may exhibit a subtle temporal delay in initial droplet formation under equivalent conditions. Here, the polymeric membrane serves as a physical barrier, impeding the initial fuel permeation but not to the extent to substantially attenuate the overall dynamics of coacervate formation and dissolution since Pluronic polymersomes are readily permeable to both fuels, PEP and G6P.^[^ ^62^ ^]^ Subsequent initial growth rate of coacervates within polymersomes is expected to be somewhat slower compared to bulk systems, as the initial diffusion rate of external fuel can affect the dissipative coacervates induced by the enzymatic reaction network within the membranous structure.^[^ ^63^, ^64^, ^65^ ^]^

To validate this hypothesis, the initial growth rate of ATP‐ and NADPH‐coacervates in polymersomes were compared with bulk analogues by monitoring the initial 20 min of coacervate formation in both cases (Figure S17, Supporting Information). During the initial phase (0–6 min), the radius of ATP‐coacervates exhibited a growth rate of ≈0.25 µm min^−1^, which escalated to 0.35 µm min^−1^ in the second phase (8–20 min). Similarly, NADPH‐coacervates grew at 0.23 µm min^−1^ and 0.42 µm min^−1^ over the respective intervals. While the growth rate was slightly slower in polymersomes compared to the bulk systems in the initial phase, the polymersomes did not undergo a lag phase after the initial phase but instead increased faster in size; this is attributed to the spatial coarsening effect ^[^ ^66^ ^]^ enabled by the confinement of polymersomes which facilitates the coalescence between droplets,^[^ ^67^, ^68^ ^]^ leading to net increase of overall coacervate droplet size. Overall, these results reveal that the enzymatic reactions can be integrated in cycles to dynamically regulate the concentration of enzymatic substrates thereby allowing realization of transient coacervates that dissipates over the course of time within polymersomes. Furthermore, even though the presence of a polymeric membrane in polymersome may impede the initial fuel permeation, it offers selective molecular transport as well as facilitated growth through confinement.

### 2.7 Mimicking Cell‐to‐Cell Communications via Intercellular Enzymatic Reactions in Polymersomes

**Figure 7:**
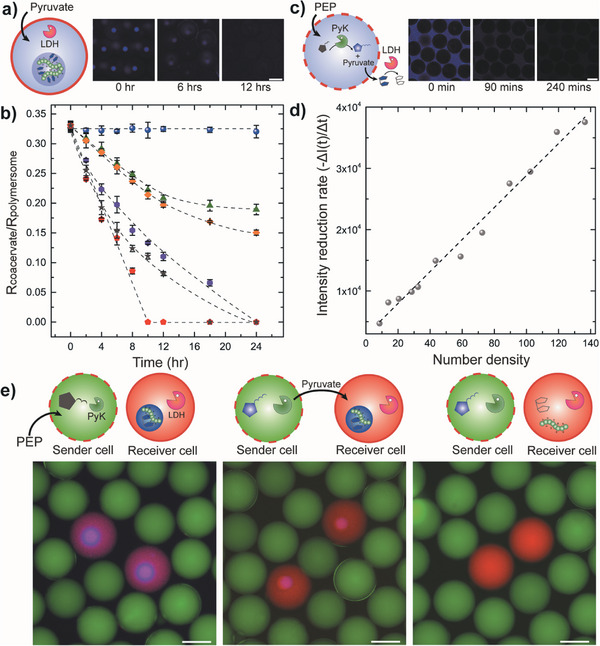
Intercellular enzymatic reactions within a heterogeneous cell community. a) Schematics and fluorescence micrographs showing the temporal dissolution of NADPH‐coacervates within PB‐PEO polymersomes upon pyruvate injection. b) Plot of temporal change in the ratio of NADPH‐coacervate to polymersome radius with variation in the concentrations of injected pyruvate. Blue circles, green triangles, orange diamonds, purple hexagons, grey stars, red pentagons each represent 0, 5‐, 10‐, 20‐, 30‐, and 40‐mm pyruvate concentration, respectively. All lines are guide‐to‐the‐eye. c) Schematics and fluorescence micrographs showing the fluorescence intensity decrease of the media containing LDH and NADPH due to PEP‐driven enzymatic reaction in Pluronic polymersomes that triggers the release of pyruvate into the media. Concentration of LDH and NADPH in the media are set as 20 U mL^−1^ and 5 mm, respectively. d) Plot of the NADPH fluorescence intensity reduction rate with variation in the number density of Pluronic polymersomes in the media. Concentration of LDH and NADPH in the media are set as 20 U mL^−1^ and 5 mm, respectively. The line is linearly fitted. e) Schematics and fluorescence micrographs before and after injection of PEP in an artificial cell community consisting of Pluronic polymersomes containing ADP, PyK and FITC‐DEX and PB‐PEO polymersomes containing pre‐formed NADPH‐coacervate, LDH and RITC‐DEX. Time‐lapse micrographs were captured at 12‐hour intervals after PEP injection. All scale bars represent 100 µm.

We also exploited the sets of enzymatic reactions discussed previously in conjunction with the different permeability characteristics of polymersomes to construct a synthetic cell‐to‐cell communicative system that allows demonstration of the dissolution of coacervate droplets through intercellular enzymatic reactions among two types of artificial cells, sender cells and receiver cells, respectively. To achieve this, we first designed the receiver cell by investigating the coacervate dissolution behavior in PB‐PEO polymersomes with respect to pyruvate concentration (**Figure** **7**a,b). Monitoring the dimensions of the NADPH‐coacervates encapsulated within PB‐PEO polymersomes with variation in the injected pyruvate concentrations revealed a direct correlation between pyruvate concentration and NADPH‐coacervate size where higher pyruvate concentrations led to faster coacervate dissolution rate. We also found that although pyruvate can permeate through the PB‐PEO membrane as discussed previously, the permeation rate tends to be rather slow, possibly due to the hydrophobic nature of PB block as well as the high portion of PEO repeating units with high electronegative oxygen ^[^ ^69^ ^]^ which can suppress the diffusion of such negatively charged molecules.

Then, we validated the potential of Pluronic polymersomes as sender cells capable of initiating the intercellular enzymatic reactions. For this purpose, we investigated their ability to metabolize externally injected PEP signal through PyK enzymatic reaction (Figure 7c and reaction I in Scheme 1). We prepared Pluronic polymersomes incorporating ADP and PyK for utilizing external PEP signal in generating byproduct pyruvate, which can readily diffuse out through the polymersome membrane into the surrounding media. In the presence of LDH and NADPH in the media, the released pyruvate can be converted into NADP^+^, resulting in reduction in blue fluorescence signal intensity of the media (Figure 7c and reaction IV in Scheme 1). As the rate at which the fluorescence intensity decreases directly depends on the amount of Pluronic polymersomes in the media, we monitored the temporal change of fluorescence intensity with variation in Pluronic polymersome number density. As anticipated, we observe a consistent trend of increase in fluorescence intensity reduction rate with increase in polymersome number density (Figure 7d and Figure S18, Supporting Information), suggesting the direct correlation between the number of sender cells and the concentration of pyruvate released to the external environment. Lastly, we combined these results to prepare a heterogeneous artificial cell community comprised of Pluronic polymersomes containing ADP, PyK and FITC‐DEX (green) and PB‐PEO polymersomes containing pre‐formed NADPH‐coacervate, LDH, and RITC‐DEX (red) each serving as the sender cell and the receiver cell, respectively. As PEP can only permeate through the Pluronic membrane, external injection of PEP in the media only affects the Pluronic polymersomes (sender cell), thereby resulting in formation of ATP in the interior. The byproduct generated by the enzymatic reaction, pyruvate, then diffuses out from the sender cell into the adjacent receiver cell, triggering the dissolution of the NADPH‐coacervate within the PB‐PEO polymersome. (Figure 7e) These results clearly reveal that the substrate‐specific response as well as the cell‐to‐cell communication in heterogeneous cell community could be recapitulated by means of signal‐driven formation and dissolution of coacervates, enabled by programming the enzymatic reaction network in these artificial cell‐like polymersomes.

## 3 Conclusions

In summary, artificial cell‐like polymersomes with distinct molecular permeability were microfluidically synthesized for fuel‐driven dissipative coacervates and mimicking cell‐cell communications. Colorimetric analysis based on enzymatic reactions were performed to assess the permeability of various enzymatic substrates through the membrane, followed by demonstration of signal‐driven formation and dissolution of ATP‐ and NADPH‐coacervates within these polymersomes. By integrating a competing reverse enzymatic reaction which depletes the active substrates into the enzymatic reaction cycle, we achieved out‐of‐equilibrium system through fuel‐driven formation of sub‐microcompartments within polymersomes followed by autonomous dissolution. As the artificial cell model outlined in this work allows spatiotemporal control of biochemical reactions as well as transient formation of synthetic membraneless organelles, we anticipate that it can be further extended to incorporate various biochemical reactions, providing a new platform to study the kinetics^[^ ^5^, ^70^, ^71^, ^72^, ^73^, ^74^, ^75^, ^76^, ^77^, ^78^, ^79^ ^]^ in cellular mimics as well as higher‐order artificial cell communities. In addition, the use of chemically versatile polymer as the building block in polymersomes offers numerous opportunities as the hydrophilic and hydrophobic building block ratio and the membrane composition can be custom synthesized to achieve desired substrate permeability ^[^ ^63^, ^77^, ^78^, ^79^ ^]^ as well as tunable molecular permeability by responding to external stimuli. Moreover, the enzymatic reaction network described in this work is not limited to polymersomes but can be also applied to liposomes, proteinosomes, and porous hydrogel microcapsules with diverse range of intrinsic permeability,^[^ ^74^, ^75^, ^76^ ^]^ opening up new possible routes to investigate library of biochemical reactions in cellular mimics.^[^ ^80^ ^]^ Overall, we envision that the concept of programmable enzymatic network in artificial cell‐like polymersome presented in this work provides a new perspective in the design of advanced artificial cell models.

## 4 Experimental Section

### Materials

Poly(ethylene glycol) (PEG, average molecular weight (MW) 8000), poly(vinyl alcohol) (PVA, 87 – 89% hydrolyzed, average MW 13000 – 23000), poly(diallyl dimethyl ammonium chloride) solution (PDDA, average MW 400000 – 500000 (high molecular weight), 20 wt.% in H_2_O), poly(allylamine hydrochloride) (PAH, average MW 17500), poly(fluorescein isothiocyanate allylamine hydrochloride) (FITC‐PAH, average MW 15000, molar ratio of FITC:PAH = 1:50), calcein, surforhodamine B, FITC‐Dextran (FITC‐DEX, MW 4000, 40000, 70000, and 150000), adenosine 5′‐diphosphate monopotassium salt dehydrate (ADP, > 95%), adenosine 5′‐triphosphate disodium salt hydrate (ATP, > 99%), phosphoenolpyruvic acid trisodium salt hydrate (PEP, > 97%), maltotriose hydrate (95%), sucrose (> 99.5%), D‐glucose (> 99.5%), β‐nicotinamide adenine dinucleotide phosphate disodium salt (NADP^+^, > 97%), β‐nicotinamide adenine dinucleotide 2′‐phosphate reduced tetrasodium salt hydrate (NADPH, > 97%), D‐glucose‐6‐phsophate disodium salt hydrate (G6P, > 98%), sodium pyruvate (> 99%), sodium lactate (> 99%), 6‐phosphogluconic acid trisodium salt (> 97%), DL‐dithiothreitol (DTT, > 98.0% for molecular biology), glutathione (GSH, > 98.0%), 2,2′‐azino‐bis(3‐ethylbenzothiazoline‐6‐sulfonic acid) diammonium salt (ABTS, > 98%), α‐glucosidase from *Saccharomyces cerevisiae*, β‐glucosidase from almonds, glucose oxidase from *Aspergillus niger*, peroxidase from horseradish, invertase from baker's yeast (S. cerevisiae), hexokinase from *Saccharomyces cerevisiae*, pyruvate kinase from rabbit muscle, glucose‐6‐phosphate dehydrogenase from *Leuconostoc mesenteroides*, L‐lactic dehydrogenase from rabbit muscle, chloroform (> 99.5%), cyclohexane (anhydrous, > 99.5%), potassium chloride (KCl, > 99.0%), magnesium chloride (MgCl_2_, > 99.0%), 4‐(2‐hydroxyethyl)piperazine‐1‐ethanesulfonic acid (HEPES, > 99.5%), n‐octadecyltrimethoxysilane, Pluronic L121 were purchased from Sigma Aldrich. Rhodamine B isothiocynate‐Dextran (RITC‐DEX, MW 70000, Invitrogen^TM^), phosphate buffered saline (PBS, pH 7.4, 1X, Gibco^TM^) were purchased from ThermoFisher. 2‐[methoxy(polyethyleneoxy)propyl]‐trimethoxy silane was purchased from Gelest, Inc. Poly(butadiene)‐b‐poly(ethylene oxide) (PB_5.2k_‐PEO_4.5k_, Sample #: P10945‐BdEO) was purchased from Polymer Source™, Inc (Canada). Glass microscope slide (76×52 mm) was purchased from LK Lab Korea. Square glass capillary and cylindrical glass capillary were purchased from Atlantic International Technology, Inc. 5 min Epoxy was purchased from Devcon.

### Estimation of Hydrophilic‐to‐Hydrophobic Volume Ratio (f Value)

The hydrophilic‐to‐hydrophobic volume ratio, also known as the *f* value, can be estimated by first calculating the average molar mass of each block comprising the block copolymer which can be determined from multiplying the average number of each block and the molecular weight of the repeat unit. It is noted that each end group of the block copolymer need to be also considered. Using the densities of the PEG and PPG reported, which is 1.13 g mL^−1^ and 1.036 g mL^−1^, respectively,^[^ ^9^ ^]^ the estimated *f* value of Pluronic L121 is 0.12. Likewise, the densities of the PB is 0.91 g mL^−1^,^[^ ^81^ ^]^ and using the molar mass ratio of the block copolymer provided, the estimated *f* value of PB‐PEO is 0.86.

### Turbidity Assay

To measure the turbidity of the coacervate suspension, the absorbance was measured using a microplate reader at a wavelength of 600 nm (Hidex Sense Microplate Reader, Hidex) after mixing the two solutions each containing anionic molecules and PDDA. Each well in the 96‐microplate contains 100 µL of total sample volumes and are read at least 3 times. Absorbance was measured after shaking the plate for 3 s at 24 °C, and the turbidity was calculated using the relation where turbidity (%) is 100(1‐10^‐Absorbance^).^[^ ^46^ ^]^

### Image Acquisitions

The production of the emulsion droplets was monitored using an inverted microscope (Eclipse Ts2, Nikon) equipped with a high‐speed camera (FASTCAM Mini UX50, Photron). Stereo micrographs were acquired using a stereoscopic zoom microscope (SMZ800N, Nikon and Capture version 4.3.0). Optical and fluorescence micrographs were obtained by using an inverted microscope (Eclipse Ti2, Nikon) equipped with a CMOS camera (Zyla 5.5, Andor). Confocal micrographs were obtained using a confocal laser scanning microscope (STELLARIS 5 Confocal Microscope, and LAS X software version 4.1.0, Leica) under the following conditions: Smart Gain 50, Smart Intensity 50, and HC PL APO CS2×10 Lens (0.40 Dry)

### Determination of the Partitioning Coefficients (K_p_) of Fluorescent Molecules in Coacervate Droplets

The extent of selective partitioning tendency of various fluorescent molecules in each coacervates system were quantified by calculating the partitioning coefficients (K_p_) defined as,^[^ ^73^ ^]^

(1) $$K_{p}=\frac{I_{\mathrm{coacervate}}-I_{\mathrm{background}}}{I_{\mathrm{dilute}\;\mathrm{phase}}-I_{\mathrm{background}}}$$

Here, the concentrations of calcein and sulforhodamine B were set at 5 µg mL^−1^ while the other fluorescent dye‐tagged dextran was set at 0.1 mg mL^−1^.

### Investigation of the correlation between the number density of polymersomes and the fluorescence intensity reduction of the external media

A solution containing ADP, NADPH, and LDH was first prepared in an Eppendorf tube (e‐tube) to measure the pyruvate‐driven NADPH fluorescence intensity reduction (reaction IV in Scheme 1). Subsequently, Pluronic polymersomes containing 20 mm ADP and 20 U mL^−1^ PyK were prepared and carefully added into the e‐tube. To initiate the enzymatic reaction (reaction I in Scheme 1), we gently inject PEP from a freshly prepared 100 mm PEP stock solution. Concentration of PEP was precisely adjusted to yield a total concentration of 20 mm in the e‐tube. LDH, ADP, and NADPH concentrations in the external media were adjusted to 20 U mL^−1^, 20 mm, and 5 mm, respectively. The total volume of the solution in the e‐tube was standardized at 200 µL. To determine the number density of polymersomes, the solution was first delicately mixed in the e‐tube using a pipette to ensure that Pluronic polymersomes are evenly distributed. After that, 2 µL of polymersome suspension was aliquoted from the e‐tube, placed it on a glass slide, and determined the number of polymersomes per microliter. Temporal change of fluorescence intensity of the media was quantified using a microplate reader (Hidex Sense Microplate Reader, Hidex).

### Statistical Analysis

Statistical analysis was performed using Microsoft Excel (Microsoft, Excel 2016) and Origin software (Origin Lab, Origin Pro 9.0). All data are presented as the mean‐standard deviations (SD), in which the error bars represent standard error of mean (SEM).

## Conflict of Interest

The authors declare no conflict of interest.

## Supporting information

Supporting Information

## Data Availability

The data that support the findings of this study are available in the supplementary material of this article.
