# Supplementary material for: Programmable Enzymatic Reaction Network in Artificial Cell‐Like Polymersomes
Source: Adv Sci (Weinh). 2024 Apr 16;11(24):2305760. doi: 10.1002/advs.202305760 (PMC11200095; doi:10.1002/advs.202305760)
Supplement: Supplementary file 1 — Supporting Information [file ADVS-11-2305760-s001.pdf]

ADVANCED SCIENCE
Open Access

Supporting Information

for *Adv. Sci.*, DOI 10.1002/advs.202305760

Programmable Enzymatic Reaction Network in Artificial Cell-Like Polymersomes

*Hanjin Seo and Hyomin Lee**

Supporting Information

Programmable Enzymatic Reaction Network in Artificial Cell-Like Polymersomes

Hanjin Seo, Hyomin Lee*

Department of Chemical Engineering, Pohang University of Science and Technology (POSTECH)

77 Cheongam-Ro, Nam-Gu, Pohang-Si, Gyeongbuk, Republic of Korea.

E-mail: hyomin@postech.ac.kr

1. Experimental Section

1.1 Preparation of PB-PEO polymersomes using a glass capillary microfluidic device

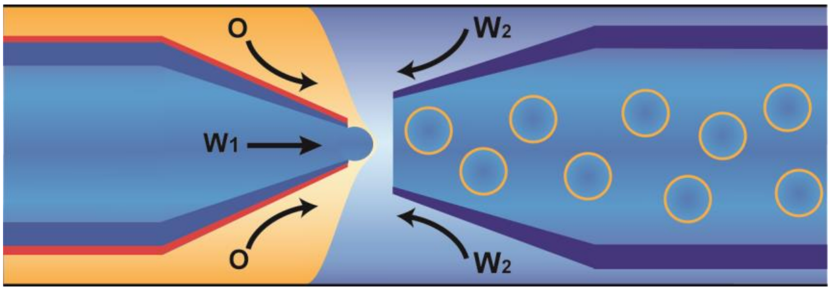

Scheme S1. Schematic illustration of the microfluidic device used to prepare $W_1/O/W_2$ double emulsion droplet templates for PB-PEO polymersomes.

To prepare PB-PEO polymersomes, a glass capillary-based microfulidic device (Scheme S1 and Figure S1) is first fabricated. The glass capillary microfluidic device comprises of a glass microscope slide, a square glass capillary, and two cylindrical capillaries. The two cylindrical capillaries were elongated using a micropipette puller (P-97, Sutter Instrument) before the surface treatment. For facile production of $W_1/O/W_2$ double emulsion droplet templates, we treated the outer surface of the injection capillary hydrophobic with n-octadecyltrimethoxysilane while the inner surface was treated with 2-[methoxy(polyethyleneoxy)propyl]-trimethoxy silane to make the surface hydrophilic as reported previously.[1] The other cylindrical capillary, the collection capillary, was treated with 2-[methoxy(polyethyleneoxy)propyl]-trimethoxy silane to make both the inner and outer surfaces hydrophilic. After the surface treatment, we tapered the tip of both capillaries with a sandpaper so that the outer diameter of the tapered collection capillary is slightly larger than that of the injection capillary. Then, a square capillary with a length of ca. 30 mm was attached to a glass microscope glass slide and the two capillaries were carefully inserted into the square capillary. The two cylindrical capillaries were precisely aligned, immobilized using a 5 min Epoxy, and the dispensing needles were attached on top for subsequent injection of each fluid into each needle. The three fluid phases comprising the droplet includes the inner aqueous phase (W_1), middle oil phase (O), and the outer aqueous phase (W_2), which were simultaneously injected through the microfluidic device as reported previously.[2] Typically, the inner aqueous phase contained 10 wt% poly(ethylene glycol) (PEG, MW 8 000) while a mixture of chloroform and cyclohexane (36:64 vt%) containing 0.1 wt% poly(butadiene)-b-poly(ethylene oxide) (PB-PEO) was used as the middle oil phase. The PEG was used in the inner aqueous phase as a crowder to increase the viscosity, enabling the double emulsion droplets to stably sink down in the container.[3] For the outer aqueous phase, 10 wt% poly(vinyl alcohol) (PVA, 87 – 89% hydrolyzed) solution was used. Here, PVA serves as a surfactant, emulsifying the middle oil phase and also helps overcoming the interfacial tension at the end of the capillary tip to produce stable W/O/W double emulsion droplet templates by increasing the viscosity of the outer aqueous phase.[4] The detailed solution composition for experiments that require the addition of substrates into the inner aqueous phase is described in the solution composition tables listed in Section 1.5. Typical flow rates of the inner, middle, and outer phase were set to 500, 3,000 and 8,000 µl/hr, respectively. The generated $W_1/O/W_2$ double emulsion droplet templates were collected in a glass chamber containing 50 mM KCl, 5 mM $MgCl_2$, and 25 mM HEPES (pH 7.4) aqueous solution to induce dewetting of the residual oil droplet.

1.2 Preparation of Pluronic polymersomes using a glass capillary microfluidic device

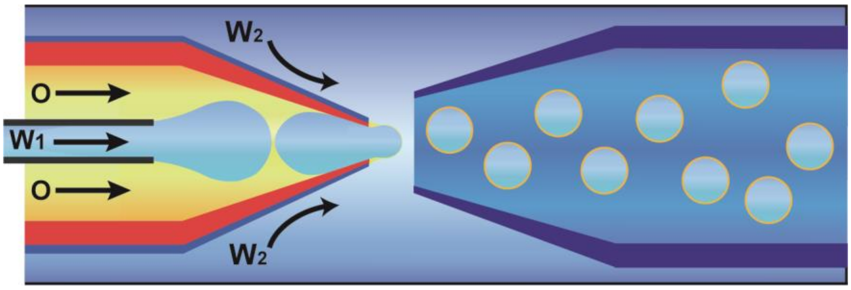

Scheme S2. Schematic illustration of the microfluidic device used to prepare $W_1/O/W_2$ ultra-thin shell double emulsion droplet templates for Pluronic polymersomes.

To prepare Pluronic polymersomes, a separate glass capillary microfulidic device comprising of three capillaries is used as reported previously (Scheme S2 and Figure S2).[5] Analogous to the aforementioned device, we assemble a glass capillary microfluidic device comprised of a glass microscope slide, a square glass capillary, and three cylindrical capillaries. Other than the injection and the collection capillary, the third cylindrical capillary, denoted as the innermost injection capillary, was elongated using a hand-held gas burner with an outer dimension smaller than the inner diameter of the unstretched end of injection capillary. Here, the inner surface of the injection capillary was treated with n-octadecyltrimethoxysilane to make the surface hydrophobic while the outer surface was treated with 2-[methoxy(polyethyleneoxy)propyl]-trimethoxy silane to make the surface hydrophilic.[5] The collection capillary was treated with 2-[methoxy(polyethyleneoxy)propyl]-trimethoxy silane to make both the inner and outer surfaces hydrophilic. After the surface treatment, the tips of the three cylindrical capillaries were additionally tapered with a sandpaper. Likewise, the resulting outer diameter of the tapered collection capillary should be slightly larger than that of the injection capillary. After insertion of the injection and collection capillaries into the square capillary, the two cylindrical capillaries were precisely aligned, fixed with 5 min Epoxy, and the innermost injection capillary was additionally inserted into the injection capillary using the identical procedure. Next, we attached the dispensing needles for subsequent injection of each fluid into each needle. The three fluid phases, the inner aqueous phase (W_1), middle oil phase (O), and the outer aqueous phase (W_2), are simultaneously injected through the dispensing needles attached to the microfluidic device. Typically, the inner aqueous phase, middle oil phase, and the outer aqueous phase is 10 wt% PEG solution, a mixture of chloroform and cyclohexane (36:64 vt%) containing 20 wt% Pluronic L121, and 10 wt% PVA solution, respectively. Similarly, the detailed solution composion is described in the solution composition tables listed in Section 1.5. The typical flow rates of the inner, middle, and the outer phase are set to 500, 800 and 6,000 μl/hr, respectively. The resulting ultra-thin shell $W_1/O/W_2$ double emulsion droplets are collected in a glass chamber containing 50 mM KCl, 5 mM $MgCl_2$, and 25 mM HEPES (pH 7.4) aqueous solution to subsequently induce dewetting of the residual oil droplet.

1.3 Preparation of PB-PEO polymersomes containing coacervates

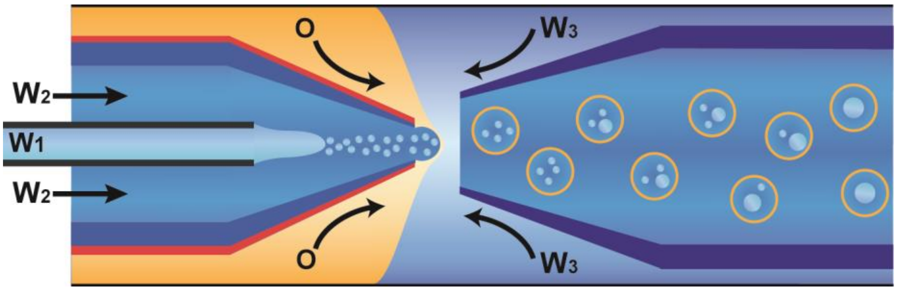

Scheme S3. Schematic illustration of the microfluidic device used to prepare $W_1/W_2/O/W_3$ triple emulsion droplet templates for PB-PEO polymersomes containing coacervates.

For cases where coacervate containing PB-PEO polymersomes need to be prepared, we assemble a separate glass capillary device extending from the one shown in section 1.2 where an additional cylindrical capillary, denoted as the innermost injection capillary, elongated using a hand-held gas burner is introduced into the device prior to attaching the dispensing needles on top. Here, water-in-water-in-oil-in-water ($W_1/W_2/O/W_3$) triple emulsion droplets are produced and co-injection of the innermost two aqueous phases result in formation of coacervates within the PB-PEO polymersomes (Scheme S3). For ATP-coacervates containing PB-PEO polymersomes, 20 mM ATP solution was injected as the innermost aqueous phase (W_1) while 80 mM PDDA solution was injected as the inner aqueous phase (W_2). On the other hand, for NADP-coacervates containing polymersomes, 10 mM NADPH solution was injected as the innermost aqueous phase and 20 mM PDDA solution as the inner aqueous phase. Other two phases (O, and W_3) were identical to the composition described in section 1.1. The typical flow rates of the innermost, inner, middle, and the outer phase are set to 500, 500, 3,000 and 6,000 µl/hr, respectively.

1.4 Preparation of Pluronic polymersomes containing coacervates

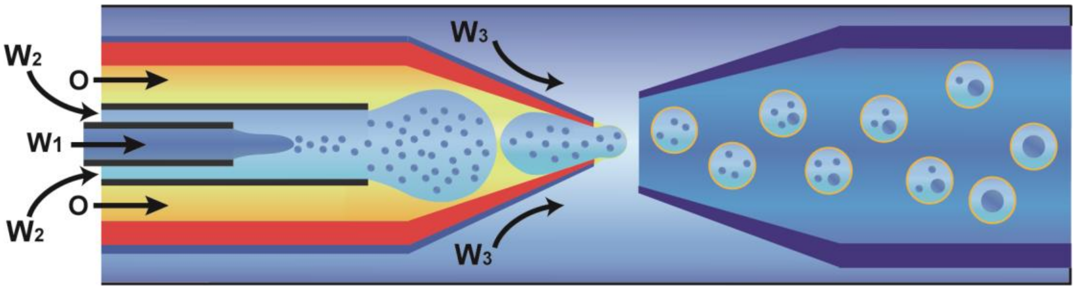

Scheme S4. Schematic illustration of the microfluidic device used to prepare $W_1/W_2/O/W_3$ ultra-thin shell triple emulsion droplet templates for Pluronic polymersomes containing coacervates.

For cases where coacervate containing Pluronic polymersomes need to be prepared, we assemble a separate glass capillary device extending from the one shown in section 1.2 in which the fourth cylindrical capillary elongated using a hand-held gas burner is additionally introduced into the previously defined as the innermost injection capillary. Here, water-in-water-in-oil-in-water ($W_1/W_2/O/W_3$) triple emulsion droplets are produced and co-injection of innermost two aqueous phases result in formation of coacervates within the Pluronic polymersomes (Scheme S4). For ATP-coacervates containing Pluronic polymersomes preparation, 20 mM ATP solution was injected as innermost aqueous phase (W_1) and 80 mM PDDA solution was injected as inner aqueous phase (W_2). For NADP-coacervates containing polymersomes, 10 mM NADPH solution was used as the innermost aqueous phase while 20 mM PDDA solution was used as the inner aqueous phase. Other two phases (O, and W_3) were identical in composition as what described in section 1.2. The typical flow rates of the innermost, inner, middle, and the outer phase are set to 400, 400, 1,000 and 6,000 μl/hr, respectively.

1.5 Solution compositions

Throughout our experiments, a consistent base composition was employed for both the inner and outer aqueous phases. Each phase contained 10 wt% PEG (inner aqueous phase) or PVA (outer aqueous phase), 50 mM KCl, 5 mM $MgCl_2$, and 25 mM HEPES (pH 7.4). The middle oil phase composition was varied based on the polymersome employed: 0.1 wt% PB-PEO or 20 wt% Pluronic L121 was dissolved in a 36:64 (v/v) chloroform:cyclohexane co-solvent mixture. All aqueous phase pH conditions were adjusted to 7.4 using 6 M NaOH or 1 M HCl. The following detailed solution compositions indicate the additionally incorporated enzymes or substrates in the inner aqueous phase and their concentrations. The outer aqueous phase composition was consistent with the base composition presented above, unless otherwise specified. Any deviations from the standard composition were noted.

[Solution composition of Figure 1]

	**Fig. 1b**	**Fig. 1c**	**Fig. 1d**	**Fig. 1e**	**Fig. 1f**	**Fig. 1g**
GOx (U mL^{-1})	10	10	10	10	10	10
HRP (U mL^{-1})	10	10	10	10	10	10
αGD (U mL^{-1})		10				
βGD (U mL^{-1})				10	10	
InV (U mL^{-1})			10			
ABTS (mM)	1	1	1	1	1	1

[Solution composition of Figure 2]

	**Pluronic polymersome**	**PB-PEO polymersome**
HK (U mL^{-1})	10	10
G6PDH (U mL^{-1})	10	10
Calcein (μg mL^{-1})		5
Sulforhodamine B (μg mL^{-1})	5	
ATP (mM)	10	10
$NADP^+$ (mM)	5	5

[Solution composition of Figure 4]

	**Fig. 4a**	**Fig. 4b**	**Fig. 4c**	**Fig. 4d**
PyK (U mL^{-1})	10			
HK (U mL^{-1})		10		
G6PDH (U mL^{-1})			10	
LDH (U mL^{-1})				10
PDDA (mM)	40	40	10	10
FITC-DEX (mg mL^{-1})	0.1	0.1		
RITC-DEX (mg mL^{-1})			0.1	0.1
ADP (mM)	10			
ATP (mM)		10		
$NADP^+$ (mM)			5	
NADPH (mM)				5

Outer aqueous phase composition: PVA 10 wt%, KCl 50 mM, $MgCl_2$ 5 mM, HEPES 25 mM, ADP 10 mM for Pluronic polymersomes in Figure 4a. In other cases, the base composition of outer aqueous phase solution was used.

[Solution composition of Figure 6]

	Fig. 6a	Fig. 6b
PyK (U mL^{-1})	20	
HK (U mL^{-1})	5	
G6PDH (U mL^{-1})		20
LDH (U mL^{-1})		10
ADP (mM)	10	
$NADP^+$ (mM)		5
PDDA (mM)	40	10
Glucose (mM)	10	
Pyruvate (mM)		50

Outer aqueous phase composition: PVA 10 wt%, KCl 50 mM, $MgCl_2$ 5 mM, HEPES 25 mM, ADP 10 mM, glucose 10 mM in Figure 6a. PVA 10 wt%, KCl 50 mM, $MgCl_2$ 5 mM, HEPES 25 mM, pyruvate 50 mM was used in Figure 6b.

[Solution composition of Figure 7]

	Fig. 7a, b	Fig. 7c, d	Fig 7e. Pluronic polymersome	Fig 7e. PB-PEO polymersome
PyK (U mL^{-1})		20	20	
LDH (U mL^{-1})	20			40
NADPH (mM)	5			5
ADP (mM)		20	40	
PDDA (mM)	10			10

Outer aqueous phase composition: PVA 10 wt%, KCl 50 mM, $MgCl_2$ 5 mM, HEPES 25 mM, ADP 10 mM for Pluronic polymersomes in Figure 7c, and d. PVA 10 wt%, KCl 50 mM, $MgCl_2$ 5 mM, HEPES 25 mM, ADP 40 mM for Pluronic polymersomes in Figure 7e. In other cases, the base composition of outer aqueous phase solution was used.

Supplementary Figures and Tables

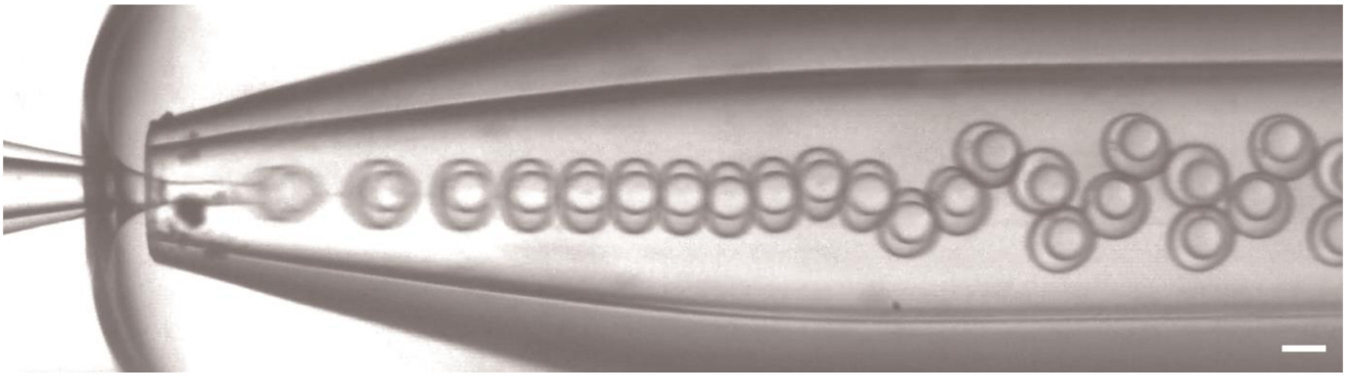

Figure S1. Optical micrograph of the glass capillary-based microfluidic device used for producing W/O/W double emulsion droplet templates. Scale bar represents 100 μm.

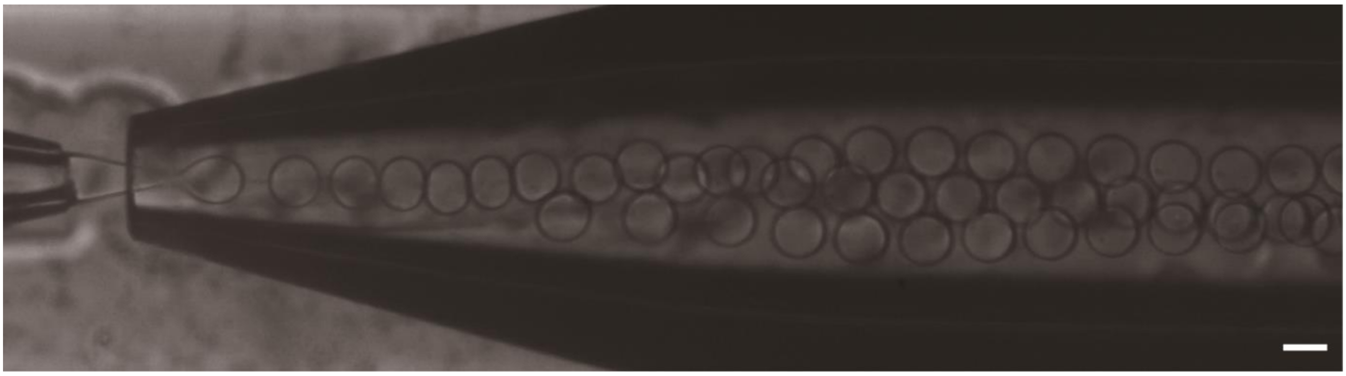

Figure S2. Optical micrograph of the glass capillary-based microfluidic device used for producing ultra-thin shell W/O/W double emulsion droplet templates. Scale bar represents 100 μm.

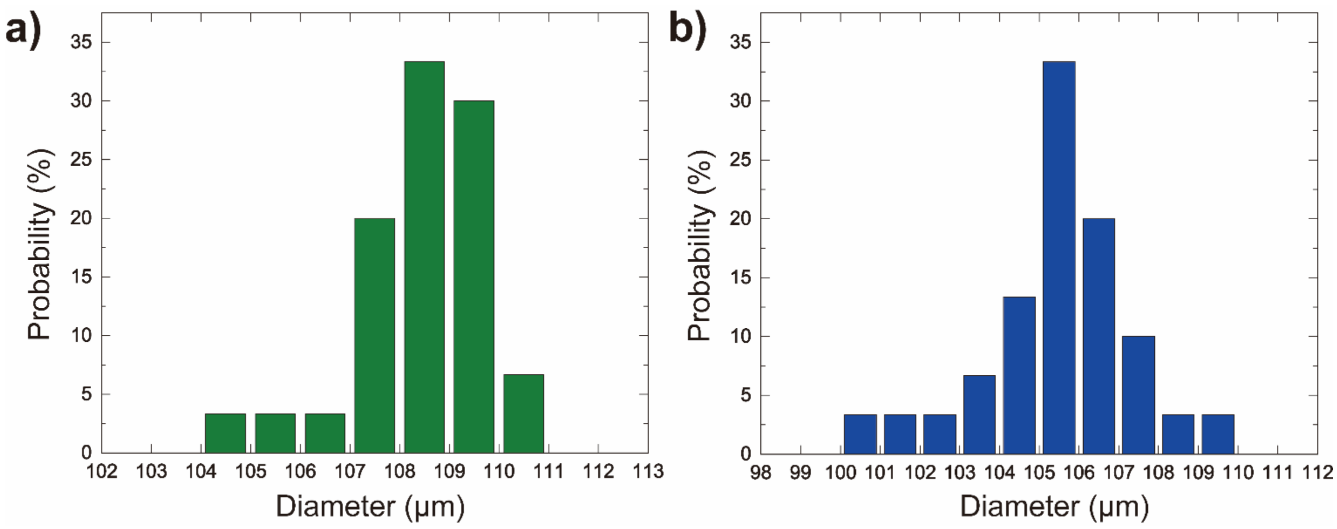

Figure S3. Size distribution on two sets of polymersomes with different membrane composition. a) PB-PEO polymersomes. b) Pluronic polymersomes. Average diameter of PB-PEO polymersomes is 108.5 ± 1.34 µm (Coefficient of variance (CV)=1.24%, n=30) while the average diameter of Pluronic polymersomes is 105.4 ± 1.91 µm (CV=1.81%, n=30).

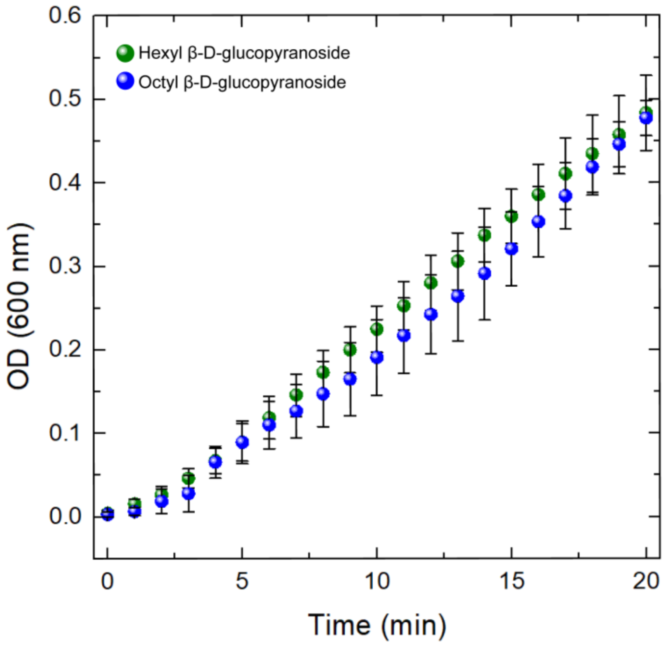

Figure S4. Plot showing the temporal optical density value (OD, 600 nm) upon monitoring the ABTS color change in reactions catalyzed by β-glucosidase (βGD), glucose oxidase (GOx), and horseradish peroxidase (HRP) in bulk (n=5). All enzyme concentrations were held constant at 10 U mL^{-1}, with 1 mM ABTS. Green and blue circles represent data points corresponding to the injection of hexyl- and octyl β-D-glucopyranoside, respectively, as substrates at a total concentration of 5 mM. Error bars represent standard deviation.

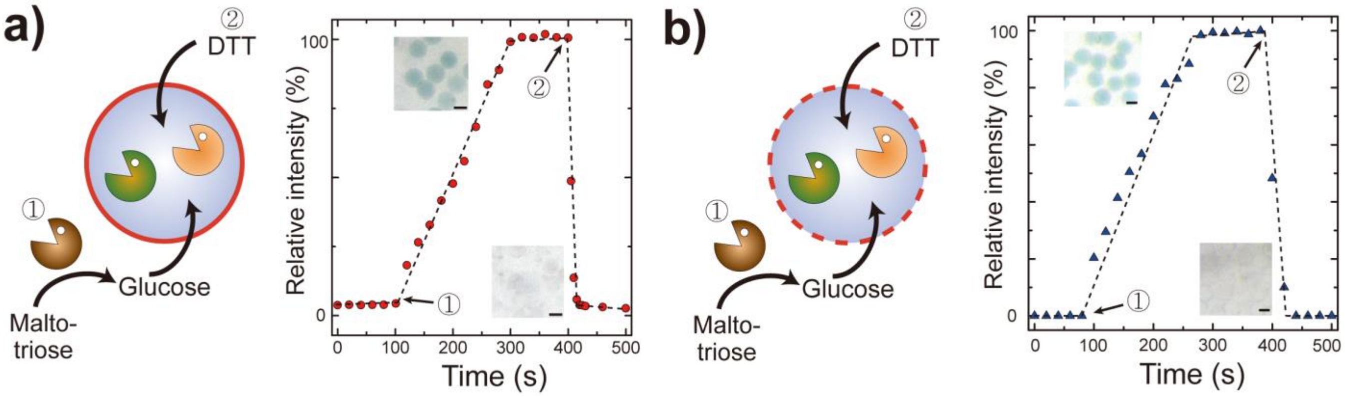

Figure S5. a-b) Schematics and the corresponding relative intensity profile plots for GOx/HRP double cascade enzymatic reaction in the presence of maltotriose upon ① injection of aGD followed by subsequent ② post-injection of a reducing agent, dithiotreitol (DTT) for both polymersomes. Concentrations of all enzymes are set at 10 U mL^{-1} and ABTS is at 1 mM. All pH conditions are 7.4. All scale bars in the inset stereo micrographs represent 100 μm.

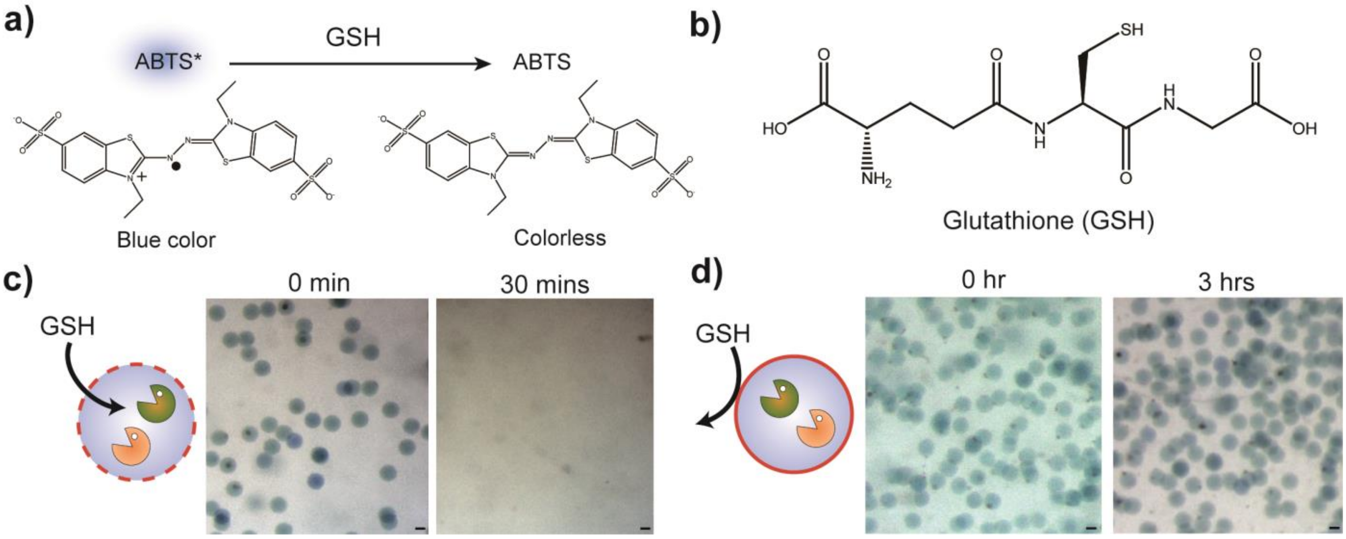

Figure S6. Colorimetric analysis based on cascade enzymatic reactions for determination of the polymersomes' permeability towards glutathione (GSH, 304 Da). a) Schematic illustration of the reaction in which GSH serve as a reducing agent and converts the ABTS* into colorless ABTS (514 Da). b) Molecular structure of GSH. c) Time lapse stereo micrographs of Pluronic polymersomes upon external injection of glucose followed by post-injection of GSH. d) Time lapse stereo micrographs of PB-PEO polymersomes upon external injection of glucose followed by post-injection of GSH. Concentrations of all enzymes are 10 U/mL, and ABTS is 1 mM. Glucose and GSH are injected to make up 50 mM and 10 mM in total concentrations. All scale bars represent 100 μm.

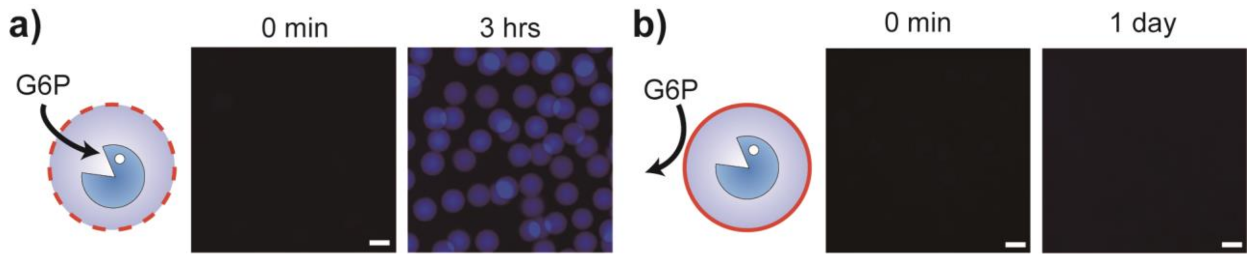

Figure S7. Colorimetric analysis based on enzymatic reaction for determination of the polymersomes' permeability towards glucose-6-phosphate (G6P, 260 Da). a) Time lapse confocal micrographs of Pluronic polymersome containing $NADP^+$ after injection of G6P. b) Time lapse confocal micrographs of PB-PEO polymersomes containing $NADP^+$ after injection of G6P. Concentration of G6PDH is 10 U mL^{-1}, and $NADP^+$ is 5 mM. G6P is injected to make up 10 mM in total concentration. All scale bars represent 100 μm.

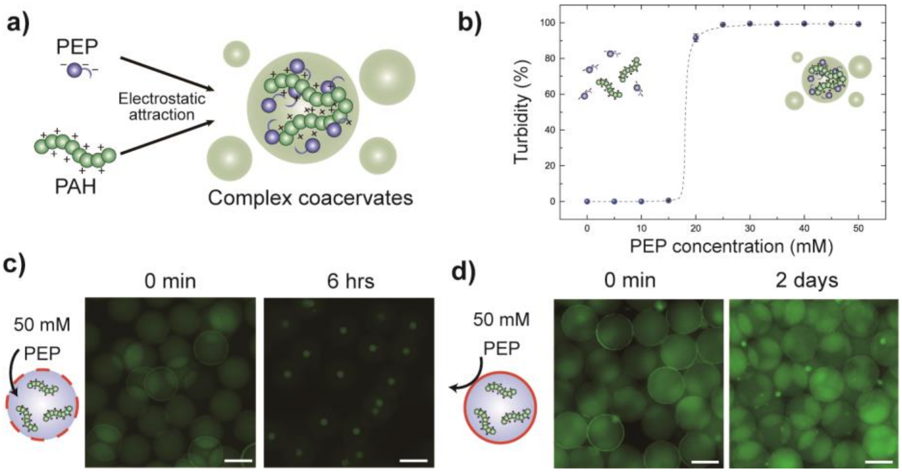

Figure S8. Determination of polymersomes' permeability towards phosphoenol pyruvate (PEP). a) Schematic illustration of the complex coacervate formation between PEP and poly(allylamine hydrochloride) (PAH). b) Plot showing the turbidity of the PEP-PAH mixed solution as a function of PEP concentration. Concentration of PAH is 10 mg/mL, and the pH is set constant at 7.4. Curve is guide-to-the-eye. c) Schematic and time lapse confocal micrgraphs of Pluronic polymersomes after injecting PEP. d) Schematic and time lapse confocal micrgraphs of PB-PEO polymersomes after injecting PEP. To visualize the coacervate formation within the polymersomes, 0.5 mg mL^{-1} FITC-PAH is included along with PAH. PEP is injected to make up 50 mM in total concentration. All scale bars represent 100 μm. Error bars indicate standard deviations.

Note. As reported earlier, we chose PAH as the positively charged polyelectrolyte to complex with externally injected anionic molecule, PEP.[2] PAH has pKa value of 9 while PEP has pKa value of 6.29 at the second ionization of the phosphoryl group.[6, 7] Therefore, at a neutral pH of 7.4 which is within an intermediate pH range of these two molecules, they electrostatically attract to form coacervate droplets. Unlike the previously reported anionic molecules with relatively larger molecular weights (such as ADP, ATP, or DNA), the coacervation of PEP with PAH occurs at a relatively high concentration regime, possibly due to its low molecular weight.[2] Under the specified buffer condition (KCl 50 mM, $MgCl_2$ 5 mM, HEPES 20 mM, pH 7.4) and PAH concentration (10 mg mL^{-1}), we found that at least 15 mM of PEP is needed to observe distinct coacervate droplets. Notably, a steep rise in solution turbidity was observed once the concentration exceeded approximately 20 mM. Based on these results, we prepared two sets of polymersomes containing 10 mg mL^{-1} PAH after which we externally introduced 50 mM of PEP in total concentration to these two types of polymersomes. We found coacervate droplets are only formed within Pluronic polymersomes unlike the PB-PEO polymersome with a homogeneous green signal in the interior which indicates no coacervate formation even 2 days after injection.

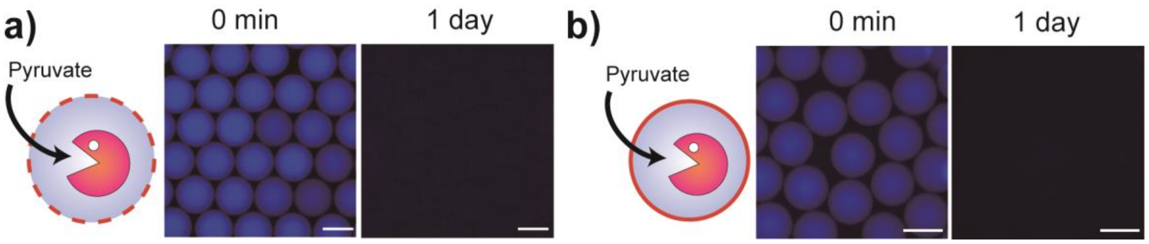

Figure S9. Colorimetric analysis based on enzymatic reaction for determination of the polymersomes' permeability towards pyruvate (88 Da). a) Time lapse confocal micrographs of Pluronic polymersomes containing NADPH after injecting pyruvate. b) Time lapse confocal micrographs of PB-PEO polymersomes containing NADPH after injecting pyruvate. Concentration of LDH is 10 U mL^{-1}, and NADPH is 1 mM. Pyruvate is injected to make up 20 mM in total concentration. All scale bars represent 100 μm.

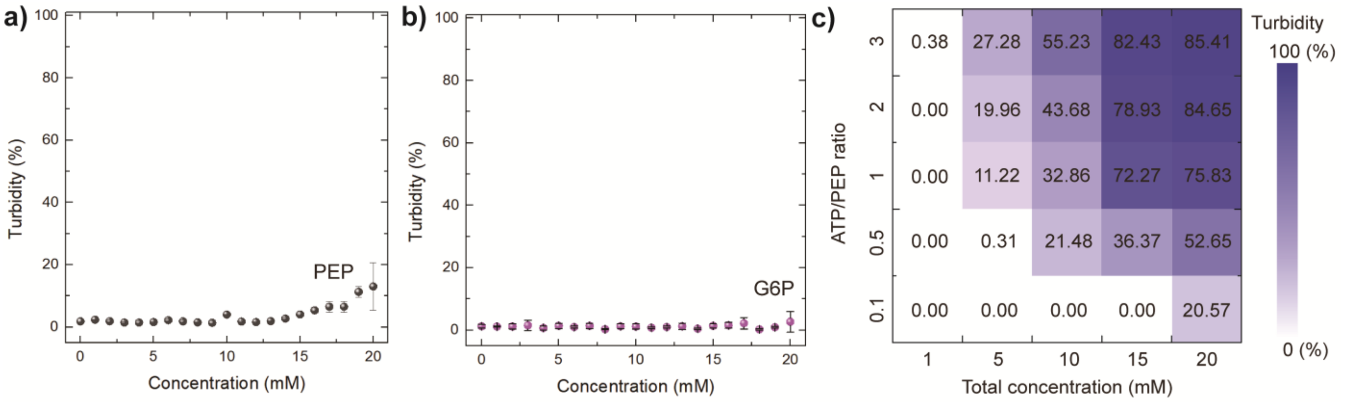

Figure S10. a) Turbidity plot by varying the concentrations of PEP at a fixed PDDA concentration of 40 mM. b) Turbidity plot by varying the concentrations of G6P at a fixed PDDA concentration of 10 mM. Error bars indicate standard deviations. c) Chart showing the turbidity change by varying the concentration ratio and total concentration of ATP and PEP.

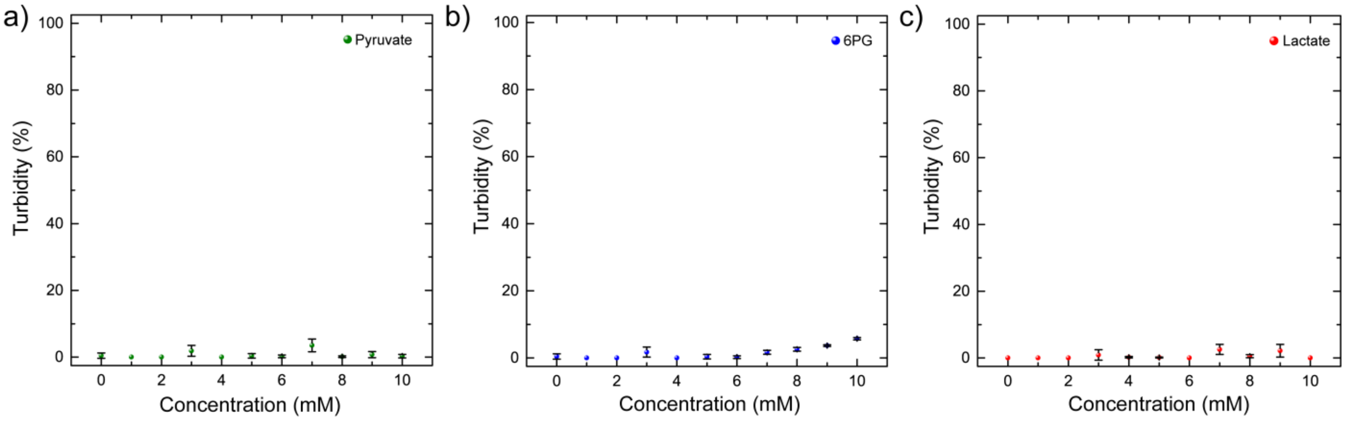

Figure S11. Plots showing the turbidity of the solution containing 10 mM PDDA and three different substrates in various concentrations, (a) pyruvate, (b) 6PG, and (c) lactate, respectively. Turbidity values were calculated by measuring the absorbance under O.D. 600 nm. All solutions were prepared at pH 7.4. Error bars represent standard deviation (n=3).

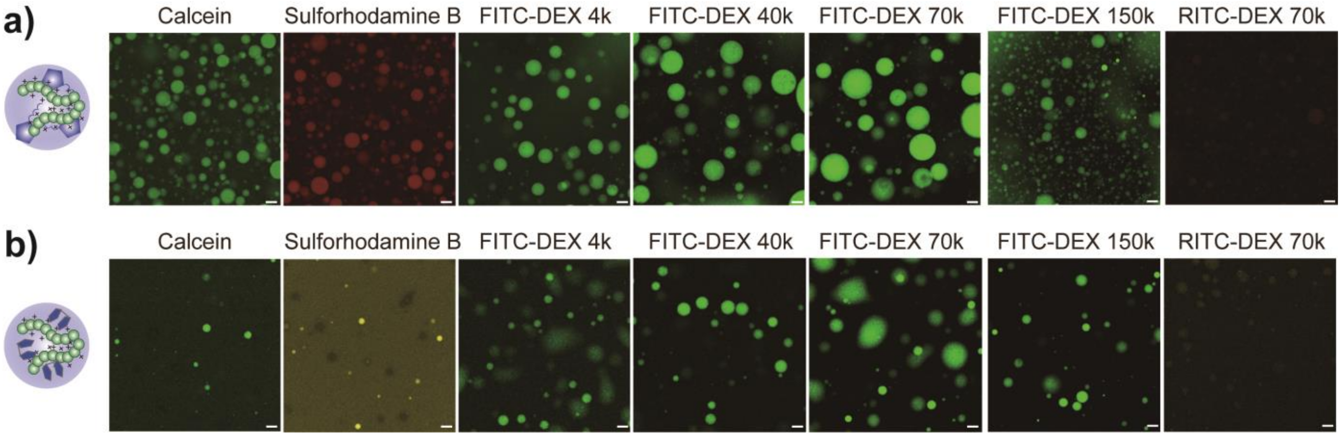

Figure S12. Sets of confocal micrographs used to determine the selective partitioning tendency of various fluorescent molecules to visualize the coacervates. a) ATP-coacervates, b) NADPH-coacervates. All scale bars represent 20 µm. For ATP-coacervates, the partitioning coefficients (K_p) values of calcein, sulforhodamine B, FITC-DEX (4, 40, 70, 150k) and RITC-DEX (70k) are 5, 5.5, 10, 32, 53, 8, and 1.5, respectively, while the K_p values for the identical set of fluorescent molecules are 8, 3.3, 9, 30, 53, 57, and 1.5, respectively, for NADPH-coacervates.

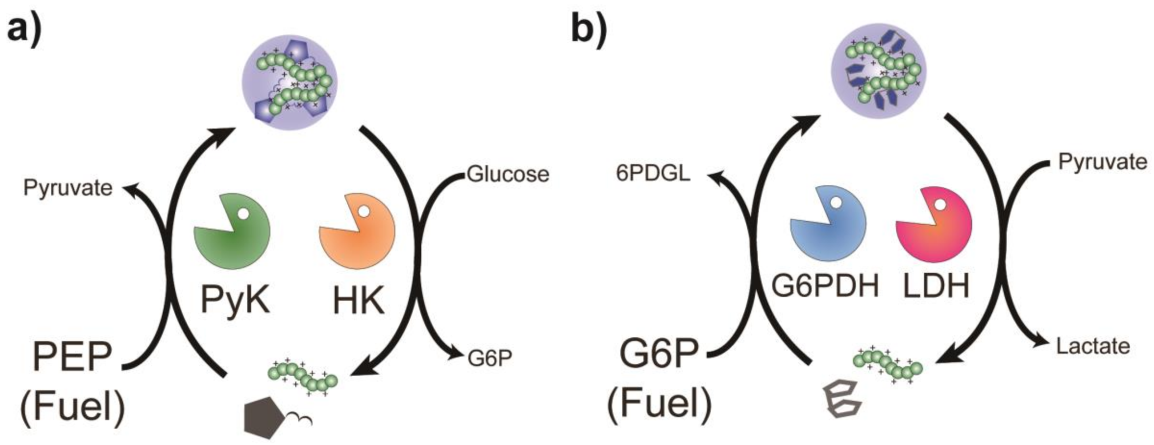

Figure S13. Schematic illustrations of the two sets of enzymatic networks used for fuel-driven dissipative coacervates. a) PEP-driven dissipative ATP-coacervates based on PyK and HK. b) G6P-driven dissipative NADPH-coacervates based on G6PDH and LDH.

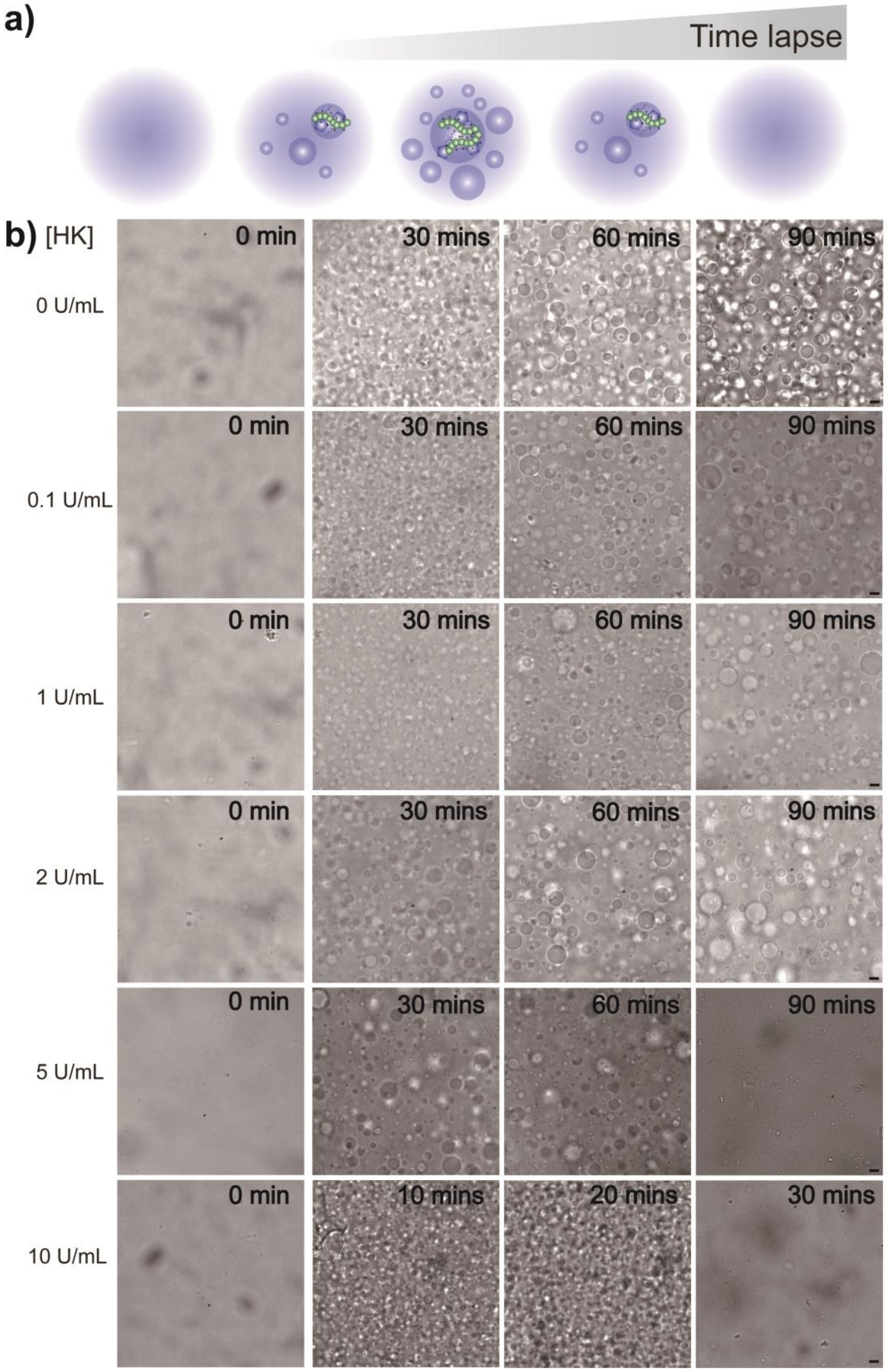

Figure S14. a) Schematic illustration of fuel-driven dissipative ATP-coacervates. b) Series of optical micrographs showing the HK concentration-dependent ATP-coacervate dynamics in bulk. All scale bars represent 10 μm, and pH is set at 7.4. Concentrations of ADP and PDDA are set as 10 mM and 40 mM, respectively. Concentrations of PyK, glucose, and PEP (fuel) are fixed at 20 U mL^{-1}, 10 mM, and 5 mM, respectively.

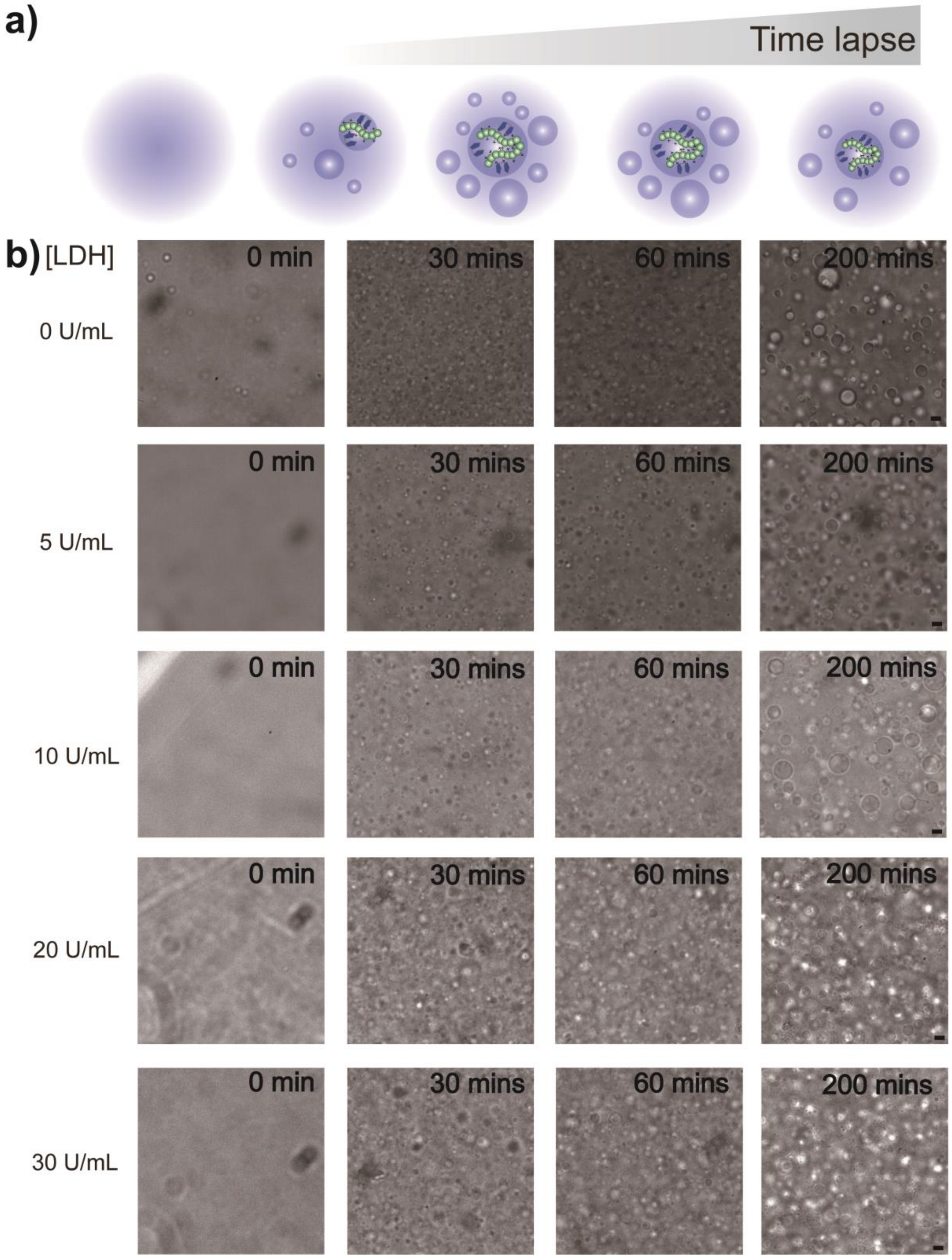

Figure S15. a) Schematics illustratng the formation and retention of NADPH-coacervates even at high LDH concentration. b) Sereis of optical micrographs showing the LDH concentration-dependent NADPH-coacervate dynamics in bulk. All scale bar represents 10 μm, and pH is set at 7.4. Concentrations of NADP+ and PDDA are 5 mM and 10 mM, respectively. Concentrations of G6PDH, pyruavte, and G6P (fuel) are fixed at 20 U mL^{-1}, 5 mM, and 3 mM, respectively.

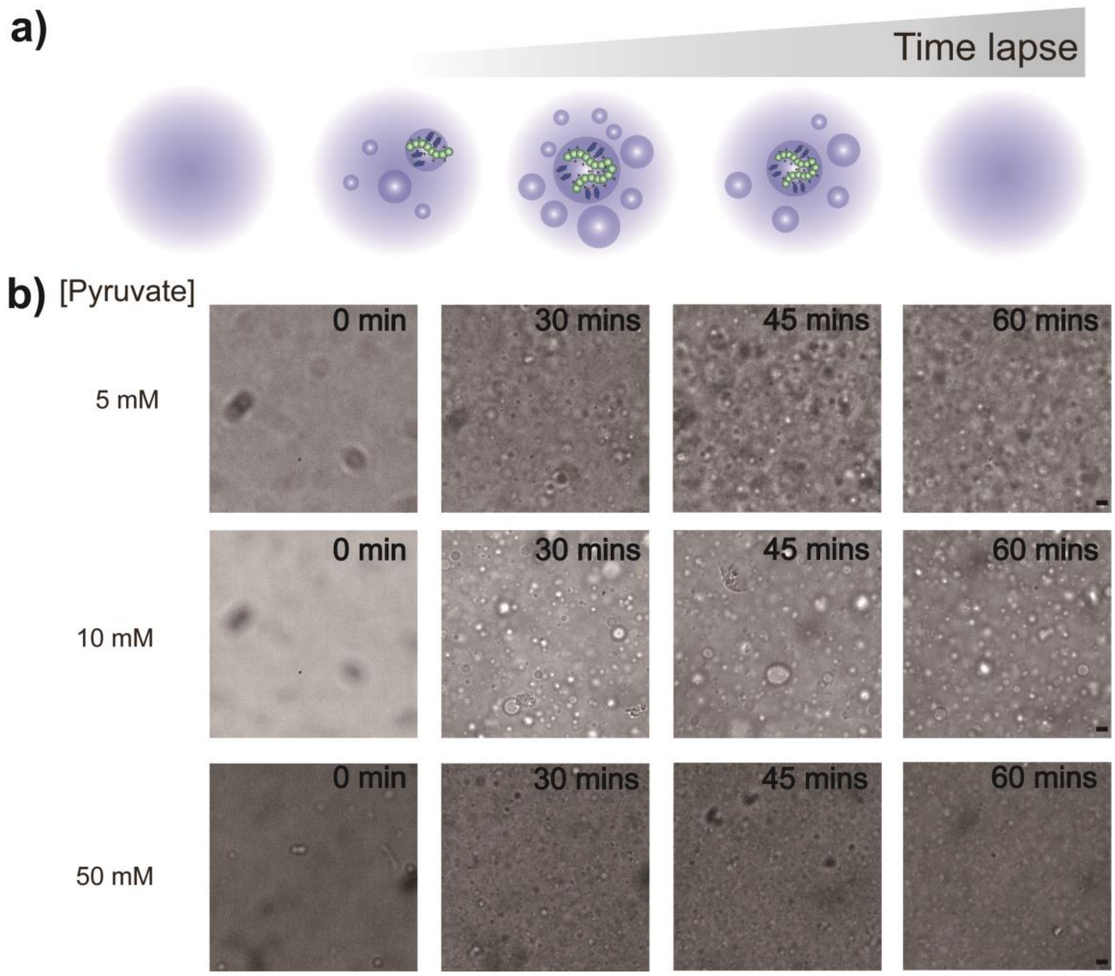

Figure S16. a) Schematic illustration of fuel-driven dissipative NADPH-coacervates. b) Series of optical micrographs showing the pyruvate concentration-dependent NADPH-coacervate dynamics. All scale bar represents 10 µm, and pH is set at 7.4. Concentrations of $NADP^+$ and PDDA are 5 mM and 10 mM, respectively. Concentrations of G6PDH, LDH, and G6P (fuel) are fixed at 20 U mL^{-1}, 10 U mL^{-1}, and 3 mM, respectively.

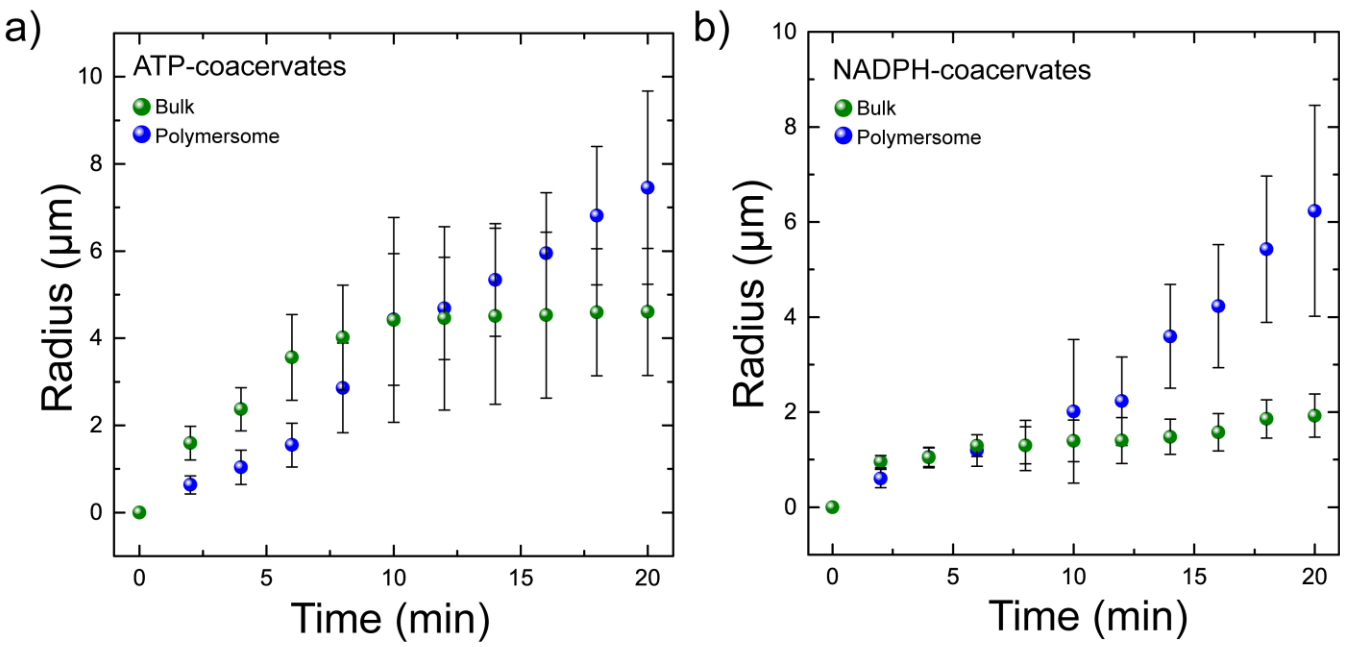

Figure S17. Plot showing the change of coacervate radius over time. a) ATP-coacervates. b) NADPH-coacervates. Blue circles indicate the size of coacervates in Pluronic polymersome (n=5) while the green circles show the coacervates in bulk (n=10).

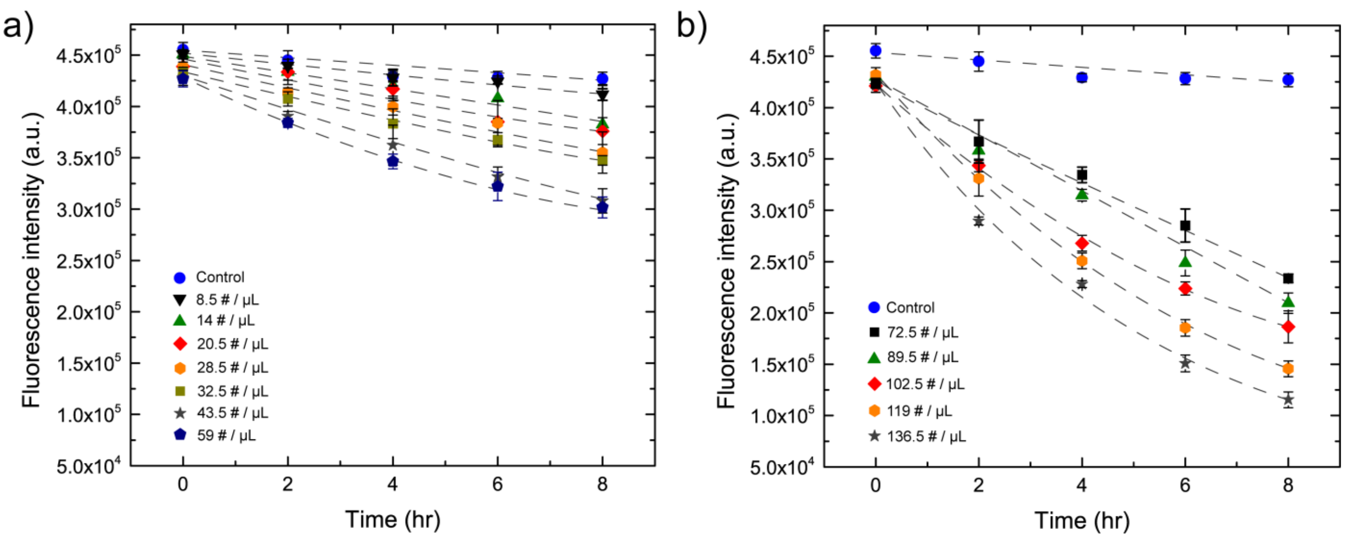

Figure S18. Plots showing the fluorescence intensity over time with variation in according to the number density of Pluronic polymersome number density. a) Plot of fluorescence intensity change for the group with low number density (n=5). b) Plot of fluorescence intensity change for the group with high number density. (n=5). Blue circles for in both plots indicate control group. The specific number density for each this condition is listed in the inset along with the corresponding symbols used in the plot. Each The inside of each polymersome contains 20 mM ADP and 20 U mL^{-1} of PyK, and the outside external media contains 20 U mL^{-1} LDH, 5 mM NADPH, and 20 mM ADP. External PEP was added as 20 mM in total media solution. Curves are guide-to-the-eye.

Table S1. List of permeable molecules for PB-PEO and Pluronic polymersomes.

Charge (pH)	Molecule (MW, charge of the component[a])	PB-PEO polymersome	Pluronic polymersome
Neutral (7.4)	Maltotriose (504 Da, 0)	Impermeable	Impermeable
	Sucrose (342 Da, 0)	Impermeable	Permeable
	Octyl β-D-glucopyranoside (292 Da, 0)	Permeable (slow)	Permeable
	Hexyl β-D-glucopyranoside (265 Da, 0)	Permeable (slow)	Permeable
	Glucose (180 Da, 0)	Permeable	Permeable
	DTT (154 Da, 0)	Permeable	Permeable
Anionic (7.4)	GSH (307 Da, -e)	Impermeable	Permeable
	G6P (260 Da, -2e)	Impermeable	Permeable
	PEP (180 Da, -3e)	Impermeable	Permeable
	Pyruvate (88 Da, -e)	Permeable	Permeable

[a]pH 7.4 condition.

References

[1] Lee, H.; Choi, C.-H.; Abbaspourrad, A.; Wesner, C.; Caggioni, M.; Zhu, T.; Nawar, S.; Weitz, D. A. Fluorocarbon Oil Reinforced Triple Emulsion Drops. *Adv. Mater*. **2016**, *28*, 8425-8430.

[2] Seo, H.; Lee, H. Spatiotemporal Control of Signal-Driven Enzymatic Reactions in Artificial Cell-Like Polymersomes. *Nat. Commun.* **2022**, *13*, 5179.

[3] Kim, S.-H.; Shum, H. C.; Kim, J. W.; Cho, J.-C.; Weitz, D. A. Multiple Polymersomes for Programmed Release of Multiple Component. *J. Am. Chem. Soc.* **2011**, *133*, 15165.

[4] Shum, H. C.; Kim, J.-W.; Weitz, D. A. Microfluidic Fabrication of Monodisperse Biocompatible and Biodegradable Polymersomes with Controlled Permeability. *J. Am. Chem. Soc.* **2008**, *130*, 9543.

[5] Seo, H.; Nam, C.; Kim, E.; Son, J.; Lee, H. Aqueous Two-Phase System (ATPS)-Based Polymersomes for Particle Isolation and Separation. *ACS Appl. Mater. Interfaces.* **2020**, *12*, 55467-55475.

[6] Nakashima, K. K.; Baaij, J. F.; Spruijt, E. Reversible Generation of Coacervate Droplets in an Enzymatic Network. *Soft Matter* **2018**, *14*, 361-367.

[7] de Silva, U. K.; Brown, J. L.; Lapitsky, Y. Poly(allylamine)/Tripolyphosphate Coacervates Enable High Loading and Multiple-Month Release of Weakly Amphiphilic Anionic Drugs: an *in vitro* Study with Ibuprofen. *RSC Adv.* **2018**, *8*, 19409-19419.
